# Changes over time in social inequality in adult self-rated health: the case of Norway 2002–2019

**DOI:** 10.1186/s12889-025-25248-w

**Published:** 2025-11-11

**Authors:** Tord Finne Vedøy, Liv Grøtvedt, Astri Syse

**Affiliations:** 1 Department of Alcohol, Tobacco and Drugs, Division of Mental and Physical Health, Norwegian Institute of Public Health, Oslo, Norway; 2 Department of Health and Inequality, Division of Mental and Physical Health, Norwegian Institute of Public Health, Oslo, Norway; 3 Department of Health and Inequality and Centre for Evaluation of Public Health Initiatives, Division of Mental and Physical Health, Norwegian Institute of Public Health, Oslo, Norway

**Keywords:** Education, Health, Income, Inequality, Norway, Occupation, Self-rated, Socioeconomic, Survey

## Abstract

**Background:**

Findings on how social inequality in self-rated health (SRH) has changed over time are not uniform. To inform public health policies aimed at reducing unwarranted social differences in health, we examine time trends in relative and absolute associations between SRH and three different socioeconomic measures using Norway as a case.

**Methods:**

Six rounds (2002–2019) of repeated national, cross-sectional, data from Statistics Norway’s Survey on Living Conditions (response rates 57–70%) were linked to register information on socioeconomic measures (education, household income and occupation). A set of logistic regression models of good SRH (GSRH) with blockwise inclusion of predictors were employed to 29,012 adults (25–66 years). Absolute and relative differences in GSRH across education, income and occupation were calculated across survey years, net of other sociodemographic characteristics and risk factors.

**Results:**

Regardless of model, the probability of reporting GSRH was high (around 80%) across sex for most years. Considering only statistically significant results, men with the short education had a 2-4% points lower probability of GSRH than men with long education, across years. The time trend in income differences was also stable, albeit the difference between the highest and lowest quintile was larger (around 7% points). Those outside the labor force were substantially less likely (23% points) to report GSRH compared to men in high skilled white-collar occupations. Among women, GSRH was 6% points lower among those with short compared to long education, and 8% points lower in the 1st compared to the 5th income quintile. Being outside the labour force was associated with a 28% points lower probability of GSRH among women. Otherwise, there were minor differences in GSRH between occupational groups. On a relative scale (percent difference), differences between socioeconomic groups were slightly more pronounced, but with similar trends for men and women.

**Conclusions:**

Irrespective of socioeconomic measure, social inequalities in SRH have remained remarkably stable over time across socioeconomic measures in Norway, in both absolute and relative terms. The national strategy may have worked to prevent increasing social inequalities in health, but system changes and further actions appear warranted to *reduce* existing differences.

**Supplementary Information:**

The online version contains supplementary material available at 10.1186/s12889-025-25248-w.

## Background

Reducing social inequality in health is an important public health aim for most developed countries, and it is also firmly grounded in the United Nations’ sustainable development goals [1]. Concerns about health inequalities are largely influenced by Wilkinson’s much debated argument, suggesting that in societies with the same level of prosperity, larger inequalities lead to poorer public health [2], with significant social and economic costs to both individuals and societies [1].

Since the 1990 s, a vast literature has described inequalities in health in terms of mortality, morbidity, and self-rated health (SRH) in European countries [3–11]. Although most studies have examined the role of education, a few studies have also included income or occupation. These older studies show that those educationally [3, 6–8, 11], financially [3, 8, 10] or vocationally [3, 9, 11] better off tend to have longer life expectancies and better health than those with fewer resources. More recent studies of changes over time across European countries confirm relatively stable and persisting social inequalities in SRH in working age populations [12–16], except for widening inequalities in some contexts associated with the Great recession, which hit Europe in 2008–2009 [17, 18]. Few studies have, however, looked jointly at education, income, and occupation. We thus aim to assess trends over time in social inequality in SRH in Norway between groups with different educations, household incomes and occupations, using six rounds of national representative survey data from 2002 to 2019, complementing existing national reviews [19, 20].

### Existing research on SRH

Three studies from Europe, also including Norway, have analyzed educational differences in SRH in the period 2000 to 2014 [15, 17, 18]. One study concluded that the recession in Europe was associated with widening educational inequalities in SRH, but this was not observed for Norway, which was categorized among the countries without ‘crisis episodes’ [17]. The other two studies found persistent, but stable, inequalities in SRH during periods of economic crises [15, 18]. Leão et al. attributed this to countries’ ability to avoid a policy of austerity [18]. Mackenbach et al., however, observed a slowdown in the decline in ’less than good’ SRH among people with both short and long education, even in countries most severely hit by the crisis [15]. A recent Norwegian study, from a regional set of surveys, show stability in absolute SRH differences by education, whereas the relative differences have increased [21]. Studies from Finland and Germany also found that educational inequalities in SRH remained fairly stable through the first decade of this century [12, 13, 22], although economic recessions and financial crises appeared to temporarily influence inequalities.

For income differences, studies tend to show that they are increasing more often than they are decreasing [8, 23, 24]. A systematic review found material factors to contribute the most in explaining socioeconomic position (SEP) inequalities in SRH, primarily due to their higher independent (direct) effect [25]. There is more debate concerning the additional shared (indirect) effect through psychosocial, behavioral and/or risk factors, such as physical inactivity, obesity and social support, although they are all clearly associated with SEP. Whereas some studies suggest that they explain relatively little of the observed variance observed for SEP inequalities [26–28], other studies suggest that they play a more important role [25, 29]. Smoking is an exception, as it appears to be a behavior that plays an important role in explaining inequalities in SRH in countries where smoking is common [30].

### Aims and research questions

This study contributes to the existing knowledge base by examining absolute and relative changes in SRH over a 20-year period, in a country with a generous social security system, using three measures of SEP, while also taking sociodemographic, lifestyle and social support factors into account. We pose the following research questions (RQs):

- RQ_1_: Have educational inequalities in SRH changed over time?
- RQ_2_: Have household income inequalities in SRH changed over time?
- RQ_3_: Have occupational inequalities in SRH changed over time?
- RQ_4_: Do the time trends differ on absolute versus relative scales?

In light of existing research, we expect the inequalities in SRH between educational groups to have remained fairly stable over time in Norway (H_1_). Furthermore, we expect to find slightly increasing income differences in SRH over the past two decades (H_2_). We do not expect notable increases in inequalties between groups related to the 2008 Great recession, as the impact of this crisis was minor in Norway due to relative high levels of prosperity and existing social welfare schemes [17, 18]. In terms of occuapational differences, our analyses are more exploratory because there is less research on occupational differences over time, and the status and work tasks associated with various occupations have changed considerably over time [31].

In terms of sex differences, we expect the associations between health and education to be similar as the educational expansion applied to both men and women, whereas the associations between health and income may increase more for women over time as the ‘male breadwinner model’ has become less common (H_3_).

Not many studies look at time trends on both absolute and relative scales, although notable exceptions exist, as stated previously. As the compositions and size of the groups in terms of education and occupation have changed considerably over time, we expect more stability over time in absolute than relative differences (H_4_).

## Data and methods

### Data

Data came from Statistics Norway’s *Survey on Living Conditions*, a repeated national, cross-sectional survey of Norwegian adults conducted every three to four years. We used data from six pooled surveys collected by Statistics Norway in 2002, 2005, 2008, 2012, 2015 and 2019. From 2015, this survey has been harmonized with the European Health Interview Survey (EHIS).^1^ These data were linked to national registry information on education, household income, and occupation. The Norwegian Agency for Shared Services in Education and Research granted ethical approval and was responsible for administration and delivery of a linked, anonymized research file.

From 2002 to 2012, data were collected primarily by telephone interviews, whereas they were collected exclusively by telephone in 2015 and 2019.^2^ An additional postal survey with a partly overlapping set of questions was conducted from 2002 to 2012. To ensure comparability over time, we opted to only include information obtained by interviews (telephone and face-to-face), and not from postal surveys.

In all surveys, respondents were selected randomly from the Norwegian population register, the overall sample size was 10 000 from 2002 to 2012, and 14 000 in 2015 and 2019. Response rates for the telephone/face-to-face interviews varied from 70% in 2002 to 57% in 2019. Details about sample size, non-response and response rates are provided in Table A1a in Additional file 1.

We only included respondents aged 25–66 years. At age 25, the majority has completed their education and begun their working careers. At age 66, the majority has not yet retired from the work force. Individual-level SEP (education, income, and occupation) is thus measured quite accurately in this age span. In total, 28 989 of 29 012 adults answered the question on self-rated health (cf. Additional file 1, Table A1b for specifics).

#### Measure of self-rated health (SRH)

SRH was assessed by the question *How do you rate your health in general?* The response categories were very good, good, fair, poor, and very poor. We dichotomized the outcome variable by combining very good and good into *good*, and fair, poor, and very poor into *less than good*, in line with previous studies [22, 32].

#### Measures of socioeconomic position (SEP)

##### Education

 was recorded using a seven-point scale denoting the respondents’ highest level of education (cf. ISCED 2011). The categories were collapsed into three groups: *Short* (no education, pre-primary, primary and lower secondary education, 0–9 years), *Medium* (upper secondary and post-secondary education, 10–12 years), and *Long* (tertiary education, 13 + years).

##### Household income

 was defined as the sum of any post-tax income for all persons in the household, divided by the number of consumption units in the household (first adult = 1, any additional person = 0.5 each) since the needs of a household do not increase proportionally to its size. This is in line with Mackenbach et al. [33]. It was grouped into quintiles by age group, sex and year, with *Q1* as the lowest and *Q5* as the highest income group.

##### Occupation 

was measured using the first digit from the International Standard Classification of Occupations (ISCO-08). The overarching categories were combined into: *High-skilled white collar* (ISCO 1,2 and 3), *Low-skilled white collar* (ISCO 4 and 5), *High-skilled blue collar* (ISCO 6 and 7), and *Low-skilled blue collar* (ISCO 8 and 9). Respondents that were outside the labor force comprise the category *Not in labor force.*^3^

#### Covariates

Sociodemographic variables include S*urvey year* (2002, 2005, 2008, 2012, 2015, and 2019), *Marital status* (married, cohabitating, or not living with a partner), *Rural/urban area of residence*, defined by number of inhabitants (> 100,000, 20,000–100,000, 2,000–19,999 and < 2,000), *Geographic region of residence* (7 categories) and *Age* as a continuous measure. *Rural/urban area* is a compound measure based on Statistic Norway’s centrality index, based on population size and nearness to larger labour markets and/or cities. Since the index has changed over time, we used a least common denominator of categories, and combined categories with few respondents.

In addition, we include a range of lifestyle variables that may help explain GSRH scores net of SEP: *Cigarettes smoking status* (do not smoke, smoke occasionally, and smoke daily), *Weekly exercise* (1+, < 1 day, and never) and *Body mass index* (BMI < 25, BMI 25–29, and BMI ≥ 30). We do the same for two measures of social support: *Number of people you can count on in case of personal problems* (3+, 1–2, and none) and having *Someone to confide in* (yes and no).

### Methods

To examine the associations between SRH and education, household income and occupation, we constructed two sex-specific sets of logistic regression models in Stata 17.0 with blockwise inclusion of covariates and listwise deletion of respondents with missing information. Model 1 regressed GSRH on all sociodemographic variables. Model 2 included the three measures of SEP (exposures) but omitted g*eographic region* due to statistical non-significance for both men and women. In Model 3 we added lifestyle measures, whereas in Model 4 we also included additional measures of social support.

The *testparm*-command was used to determine inclusion of variables in subsequent models, cf. Additional file 2 which shows the odds ratios (ORs) from all four models. In line with our conceptual framework, both BIC and AIC strongly suggest that Model 4 (including lifestyle behaviors and/or characteristics) fitted the data best. Using estimates from this model, we calculated predicted probabilities of good self-reported health (GSRH) and marginal effects for education, income, and occupation in absolute (*dydx*) and relative (*eydx*) terms^4^ across survey years, cf. Additional file 3 for estimates.

To examine changes over time in the association between SEP and GSRH, we ran three additional models including an interaction between year and each of the three exposure variables, cf. Additional file 4.

Lastly, we calculated the relative (RII) and slope index of inequality (SII) from a set of gender specific models including the interaction between survey year and sex- and year-specific *ridit scorings*. In short, a *ridit* analysis transforms a categorial variable (for example *Education*) into a numeric variable from which one can calculate RII (the relative increase from the lowest to the highest value) and SII (the absolute difference between the lowest to the highest value), cf. Additional file 5 for further explanation and results. Fig. 2 and A5a/A5b in Additional file 5 were created using the *coefplot*-command [34].

## Results

### Descriptive statistics

The distributions of respondents across all variables by sex are presented in Table 1. A large majority of women and men (80 and 82%) rated their health as very good or good. Mean age of the sample was 45 years and there was an even distribution of men and women and respondents from urban and rural regions. Three quarters of the sample was either married or had a cohabiting partner. Substantial shares had a long education (34% men and 44% women) and near half had a high-skilled white-collar occupation. Around 70% of men and women reported no daily or occasional smoking. Among men, 69% reported exercising at least once a week and 40% reported a BMI < 25. Among women, the corresponding values were higher for both exercise (76%) and BMI (60%). Large shares (> 76%) had three or more people to turn to in case of personal problems, and the vast majority (> 95%) reported that they had someone to confide in.

**Table 1:** Distributions of variables across sex^a^

		Men			Women		
		Sample *N*	%	SD	Sample *N*	%	SD
Good self-rated health (GSRH)	Yes	14 570	81.8	38.6	14 419	80.1	39.9
	No	14 570	18.2	38.6	14 419	19.9	39.9
Year	2002	14 586	17.2	37.7	14 426	16.7	37.3
	2005	14 586	16.7	37.3	14 426	16.7	37.3
	2008	14 586	15.7	36.3	14 426	16.1	36.8
	2012	14 586	13.5	34.1	14 426	13.2	33.8
	2015	14 586	18.9	39.2	14 426	18.9	39.1
	2019	14 586	18.1	38.5	14 426	18.4	38.7
Age	Mean (not percent)	14 586	45.6	11.7	14 426	45.4	11.7
Marital status	Married	14 578	54.2	49.8	14 422	54.6	49.8
	Cohabiting	14 578	19.5	39.6	14 422	18.8	39.1
	No partner	14 578	26.3	44.1	14 422	26.6	44.2
Rural/urban area^b^	> 100 000	13 126	26.7	44.2	13 064	27.7	44.8
	20 000–100 000	13 126	21.7	41.2	13 064	21.2	40.9
	2 000–19 999	13 126	27.7	44.7	13 064	28.2	45.0
	< 2 000	13 126	23.9	42.7	13 064	22.8	42.0
Adjusted household income quintiles	q5	14 180	20.2	40.2	14 137	20.6	40.5
	q4	14 180	20.3	40.2	14 137	20.3	40.2
	q3	14 180	20.2	40.2	14 137	20.4	40.3
	q2	14 180	19.5	39.6	14 137	19.2	39.4
	q1	14 180	19.7	39.8	14 137	19.4	39.6
Education	Long	14 226	34.3	47.5	14 060	44.1	49.7
	Medium	14 226	51.2	50.0	14 060	41.9	49.3
	Short	14 226	14.5	35.2	14 060	13.9	34.6
Occupation/Employment	High-skilled white collar	14 548	47.0	49.9	14 405	45.9	49.8
	Low-skilled white collar	14 548	11.4	31.7	14 405	27.2	44.5
	High-skilled blue collar	14 548	17.2	37.7	14 405	2.4	15.2
	Low-skilled blue collar	14 548	10.4	30.6	14 405	4.6	21.0
	Outside labor force	14 548	14.1	34.8	14 405	19.9	39.9
Smoking status	Do not smoke	14 547	69.3	46.1	14 400	71.4	45.2
	Occasionally	14 547	9.6	29.5	14 400	8.4	27.8
	Daily	14 547	21.1	40.8	14 400	20.2	40.2
Exercise	≥ 1 time/week	14 545	68.6	46.4	14 395	76.3	42.5
	< 1 time/week	14 545	14.7	35.4	14 395	11.5	31.9
	Never	14 545	16.7	37.3	14 395	12.2	32.7
Body Mass Index	Normal/underweight (BMI < 25)	14 473	39.7	48.9	14 052	59.9	49.0
	Overweight (BMI 25–29)	14 473	46.9	49.9	14 052	29.3	45.5
	Obese (BMI ≥ 30)	14 473	13.4	34.1	14 052	10.7	31.0
Help in case of personal trouble?	3+ people	14 469	76.4	42.5	14 351	82.6	37.9
	1 or 2	14 469	22.1	41.5	14 351	16.4	37.1
	None	14 469	1.6	12.4	14 351	1.0	9.8
Someone to confide in?	Yes	14 522	95.8	20.0	14 392	97.8	14.7
	No	14 522	4.2	20.0	14 392	2.2	14.7

^a^Distributions by geographic region are not shown (available on request)

^b^In addition to number of inhabitants, categorizations were also based on closeness to larger cities and labour market regions

When we examined descriptive changes in distributions across survey years for both sexes combined (cf. Additional file 1, Figure A1b), we found that the share reporting GSRH was stable (78–82%). Among the SEP exposure variables, the largest change was observed for education, where the share reporting the longest education increased by 15% points (pp) from 2002 to 2019. Among the covariates, the largest positive changes were observed for never exercising (−12 pp) and daily smoking (−18 pp). Obesity, on the other hand, increased (+ 7 pp), as did the share without a partner (+ 6 pp).

### Modeled results

**Fig. 1:**
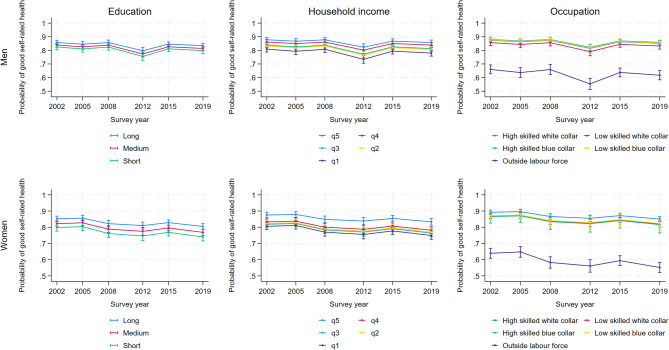
Probability of reporting good self-rated health among men and women, by education, income and occupation, Norway 2002-2019

Our main results are portrayed in Fig. 1, which shows probabilities of GSRH from Model 4 (our final model) across education, income, and occupation. In short, both male and female respondents with long education or in the highest income quintile, had a generally higher probability of GSRH compared to respondents with short education or in the lowest income quintile (4 to 6% points lower when comparing shortest and longest education, and 7 to 8% points lower when comparing lowest and highest income quintile). The differences between groups were, however, relatively minor, and GSRH was generally stable and above 0.8 (80%) throughout the period. This was observed regardless of the statistical model applied (cf. Additional file 2). Additional file 3 visualizes the corresponding absolute (pp, *dydx*) and relative (%, *eydx*) changes in estimates.

For different occupations, the distribution of GSRH among those who participated in the labor market was comparable to those observed for long education and high income. However, respondents who were outside the labor force reported much lower probabilities of GSRH (around 0.6 or 60%), and the probabilities were lower in 2019 compared to 2022 (9% points lower among women (*p* < 0.01) and 4% points lower among men (*p* < 0.05)). Models with interaction terms between year and socioeconomic variables supported these findings across the three SEP measures (cf. Additional file 4).

**Fig. 2:**
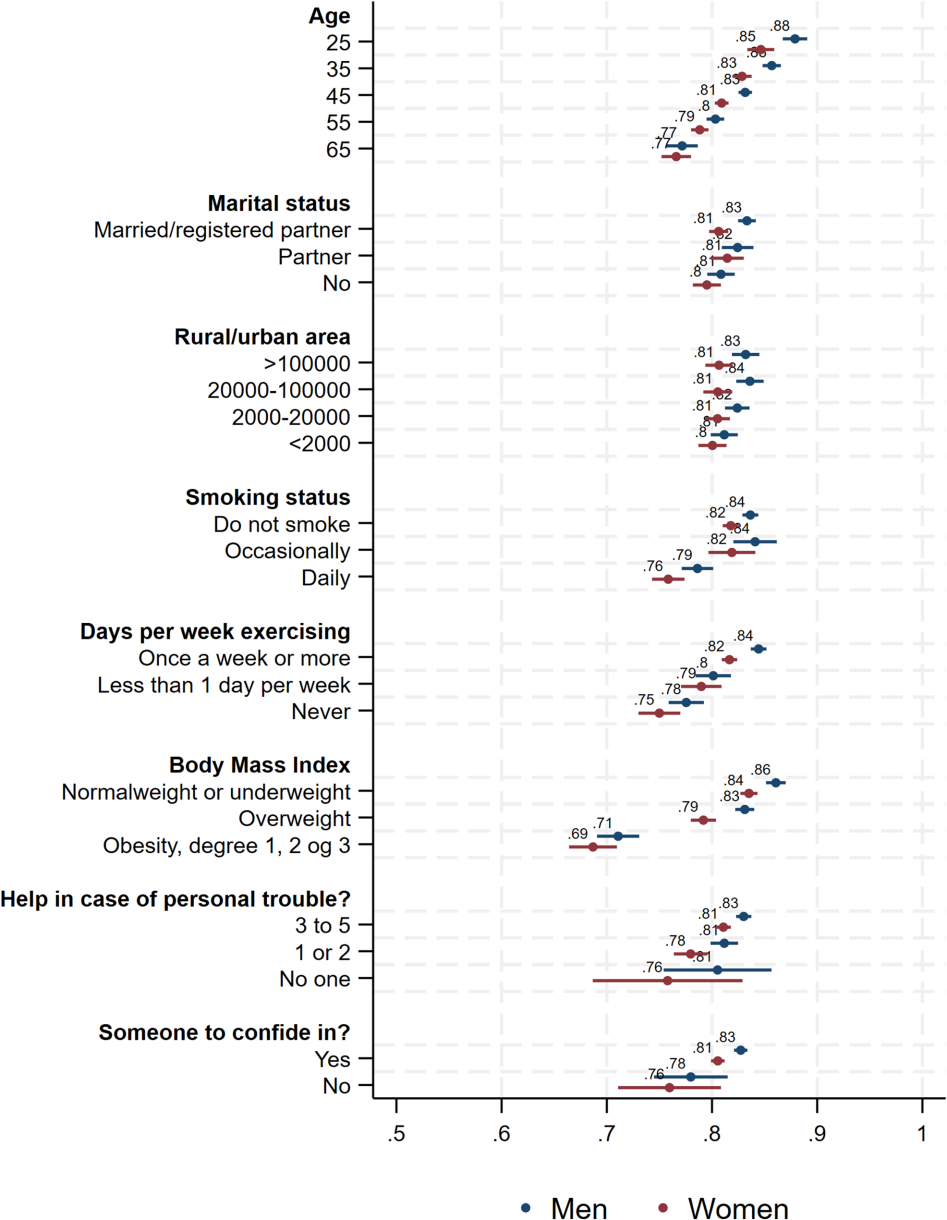
Predicted probabilities of reporting good self-rated health at each value of the covariates from Model 4 (survey year and SEP measures omitted)

The probabilities of reporting GSRH for all covariates in the final model are shown in Fig. 2. The results show that GSRH did not vary substantially with marital status or urbanity of place of residence. However, GSRH decreased with age and was relatively strongly associated with lifestyle variables (smoking, exercise, and BMI) in expected directions. The probability of reporting GSRH was, for instance, 0.86 and 0.84 among men and women with BMI < 25, and 0.71 and 0.69 among those with BMI ≥ 30.

Results from models using the Relative Index of Inequality (RII) and Slope Index of Inequality (SII) are shown in Additional file 5. These results (Figure A5a and A5b) were stable, irrespective of inequality measure used, and largely comparable to the results from the main model (Model 4) shown in Fig. 1.

## Discussion

Norway is a welfare state, with citizens benefitting from a range of health and welfare services, including income security [35]. Strong public health regulations are also reflected in the Norwegian Public Health Act, first adopted in 2011 [36]. Similar national egalitarian health programs have also been launched in other European countries, like the UK, the Netherlands, Sweden and Finland [12].

In a nationally representative survey sample with solid response rates and linked to sociodemographic register data, we found stable trends over time in self-rated health inequalities in both absolute and relative terms. Irrespective of SEP, the vast majority reported good health across most survey years. In both absolute and relative terms, the social gradient in health persisted for both education and income, in line with H_1_ but contrary to H_2_. Furthermore, we did not find significant differences in the association between health and income between men and women, and thus no support for H_3_. Lastly, the change over time was not substantially different on absolute and relative scales, contrary to H_4_. We observed minor differences between occupational groups among the employed, whereas those outside the labor force reported considerable worse health.

Comparing our findings with those of others, we see that the results for education compare well with those reported by others, cf. for instance, Mackenbach et al. [15] and van der Wel et al. [17]. Increases in inequalities in health have been related to lack of health improvement among those with short education [17]. We did not corroborate this finding, perhaps due to the inclusion of health behaviors and risk factors known to be more prevalent in this group [37]. A recent German study found stability in educational inequality in both absolute and relative terms among older men of working age (50–64), whereas an increase in absolute terms was observed for women [16]. The authors note that findings on inequalities may be life-stage specific, and that a further disentanglement of period and cohort effects may be warranted [16]. Our findings were also contrary to those observed in a large European study covering 20 years (Norway not included), which found decreasing trends in the prevalence of less than good health and increasing relative differences by education and occupation (but stability in absolute terms) in many of the 17 studied countries [11].

Our results for income align well with one German study, which found education and income inequalities in SRH to be considerable and persisting from 2003 to 2012 [22]. Our findings run contrary to an earlier German study, which found income-related inequalities in SRH to roughly double from 1994 to 2011 [38]. This is also underscored in a systematic review, which underscores that “research has shown that health inequalities by income and occupation status increased over the last few decades” [25]. Thus, also for the various occupational groups, we found smaller differences between those employed than previously reported [25]. Not surprisingly, the economically inactive in our study reported lower levels of GSRH than those who were employed. This is in line with results from a recent Canadian study [39], which suggest widening relative and absolute health inequalities between the employed and unemployed over time, primarily due to an increase in the prevalence of poor health among the unemployed. The same trend was observed in a EU-SILC study [40], which showed a steep decline in reports of good health among the unemployed. In our study, the negative trend was statistically significant and was more pronounced and declined more over time for women than men, contrary to what has been reported previously [40]. Developments in characteristics associated with health cannot, however, shed light on why the health inequalities between the employed and unemployed have widened over time. It should be noted that there is a difference between unemployment and non-employment. In our study, the economically inactive comprised *all* individuals aged 25 to 66 years outside the labor force, irrespective of cause (e.g., unemployed, students, health-related pensioners, home makers, etc.). As they comprised quite a large share of respondents (14% of men and 20% of women), further research directed at this group appears warranted given the size of the group and the decline in GSRH over time, especially among women.

Previous studies have shown that changes in trust, social relationships, and experiences of economic hardship account for much of the reported increase in SRH inequality, and inequalities have generally been found to be more pronounced in countries experiencing simultaneous austerity and economic recession [17], with which Norway has limited experience. Circumstances in which people live and work are, however, important for communicable as well as for non-communicable diseases also in welfare states [19]. Whereas the current trends in SRH appear relatively stable, it will be interesting to see if the recent trends of increased social inequalities after chronic illness and functional limitations in this millennium [41, 42] will translate into greater inequality also in SRH in the years to come.

### Limitations and strengths

The cross-sectional survey design only enables us to report on associations. Other data and designs are needed to examine possible causal effects. Furthermore, our outcome measure is self-reported. Although SRH is a latent concept, resulting from “a cognitive process that is inherently subjective and contextual” [43], it has been found to be a valid umbrella measure encompassing both physiological and mental health across European countries, reflecting inequalities in mortality as well as bodily and mental functions between different social groups [25, 28, 44]. As such, it is considered a useful indicator for assessing inequalities in health [45]. A more problematic issue is self-selection, an inherent problem in all surveys, as those who choose to take part are likely to differ from those who refrain. Although our data were balanced in terms of age and sex, persons with fewer resources in terms of education, income and occupation were slightly underrepresented [46]. This might have led to an underestimation of the associations between GSRH and SEP, and due to declining response rates over time [46] this might be more of an issue in recent survey rounds than in earlier ones.

Most studies on socioeconomic health differences use education as the (only) exposure variable. To achieve a more comprehensive understanding and interpretation of the development of health differences over time, we used education, income quintiles and occupation as exposure variables in our analyses. Although these measures are intrinsically linked, they were not highly correlated^5^, and they each contribute to the overall understanding of social inequalities in health. Furthermore, all these measures were obtained from registers, and information bias should thus be minimal. The education variable may, however, slightly underestimate attained educational level for younger persons, as some continue in education after age 25. We therefore re-ran all analyses excluding those younger than 40 years, and the results were similar (not shown).

Income represents disposable income after tax, including all income sources, and the measure is thus likely to accurately reflect respondents’ economic situation. Another strength is that we have measured differences in income according to rank, thus contributing to cross-national discussions on this topic. Norway is, however, an affluent country with relatively low levels of economic inequality^6^. Although we lack measures of capital income and/or homeowner information in our data, the high degree of correlation between incomes and wealth in Norway [47] makes it likely that the overall conclusions in terms of material resources remain valid. Economically inactive cannot be classified based on their main occupation, and we thus opted to group them separately. As their results were markedly different from other occupational groups, this appeared to be an appropriate choice. Further research directed at this group appears warranted given the marked share men and (especially) women outside the labor force comprise and their suboptimal (and among women, declining) health.

Whether our findings apply to other contexts with different levels of affluence and inequality warrants further research, as studies have shown that experience of economic hardship might contribute to increased SRH inequality, especially in countries experiencing simultaneous austerity and economic recession [15]. Lastly, unlike many previous studies, we were able to account for changes in health behaviors and risk factors. On the one hand, this might have resulted in lower levels of both absolute and relative inequalities by SEP, because lifestyle variables may function as mediators between SEP and SRH. Future studies should examine such pathways between SEP and SRH more closely. On the other hand, the inclusion of these variables enhanced the predictive capacity of the model, and since there has been pronounced changes over time in both health behaviors (smoking and exercise), risk factors (BMI) and other confounding variables (e.g., partner status), our ability to include these measures in the final models might provide a clearer association between SEP and self-rated health.

## Conclusions and the way forward

Irrespective of socioeconomic measure, social inequalities in SRH have remained remarkably stable over time in Norway, in both absolute and relative terms. The Norwegian national strategy may have worked to prevent increasing social inequalities in health, although similar findings are observed also in countries without such explicit strategies [11]. Hu et al. [11] observed that countries with encouraging and significantly decreasing trends in social inequalities in SRH, such as Italy, employed similar national level efforts to reduce inequalities as, for instance, England and Scotland, but with different results. Consequently, the evidence base for the effects of national policies to tackle health inequalities appears relatively weak, although it is not clear how the situation would present without such policies in place. Nevertheless, new actions and fundamental system changes appear warranted if the aim is to *reduce* existing differences. Actions on the social determinants of health is necessary to improve health, but it is also an issue of social justice [19]. How such actions should be designed and what they should entail remains, however, unclear. A Norwegian study underscores the problematic divide between public health strategies at local and national levels, in that although many municipalities focus on lifestyle and healthcare related measures, only a few acknowledge the social determinants of health and have implemented health impact assessments and health overviews to this end [48]. The authors thus argue for concerted multilevel actions to reduce health inequalities, implying that national and local approaches to a larger extent need to complement each other. Although the social protection offered by the Norwegian welfare system [18] might be sufficient to hinder an expansion of social inequalities in health, new actions and fundamental system changes appear warranted if the aim is to *reduce e*xisting differences.

## Supplementary Information

Additional file 1. Sample size, response rates and descriptive changes over time for the pooled sample. Table A1a. Sample size and response rates in interview surveys of living conditions 2002-2019. Table A1b. Distributions of variables in Model 4, percent (%) and number of respondents (n), 2002-2019, men and women combined.

Additional file 2. Model choice. Table A2a. Odds ratios (OR), standard errors (SE) and p-values (p) from Model 1 to Model 4, men. Table A2b. Odds ratios (OR), standard errors (SE) and p-values (p) from Model 1 to Model 4, women. Figure A2a. Probability of good self-rated health among men and women, Norway 2002-2019. Estimates from all models.

Additional file 3. Differences over time in percentage points and percentages. Figure A3a. Absolute (percentage points) differences (dydx) in reports of good health among men and women, by education, income and occupation, Norway 2002-2019. Figure A3b. Relative (percent) differences (eydx) in reports of good health among men and women, by education, income and occupation, Norway 2002-2019. Table A3a. Marginal mean probabilities (Margin), marginal effects (dydx), semi-elasticities (eydx), standard errors (se) and p-values (p) from Model 4 for men and women.

Additional file 4. Effect modification. Figure A4a. Probability of reporting good health among men and women, by education, income and occupation, Norway 2002-2019, from models including interaction terms between the relevant exposure variable and year. Figure A4b. Absolute (percentage point) difference (dydx) over time in reports of good health among men and women, by education, income and occupation, Norway 2002-2019, from models including interaction terms between the relevant exposure variable and year. Figure A4c. Relative (percent) difference (eydx) over time in reports of good health among men and women, by education, income and occupation, Norway 2002-2019, from models including interaction terms between the relevant exposure variable and year. Table A4a. Odds ratio (OR) estimates from 3 different models including an interaction term between year and the exposure variables.

Additional file 5. Relative Index of Inequality (RII) and Slope Index of Inequality (SII). Figure A5a. Relative index of inequality (RII) over time in reports of good health from a modified Model 4, based on ridits for education, income and occupation, Norway 2002-2019. Figure A5b. Slope index of inequality (SII) over time in reports of good health from Model 4 (men) and Model 3 (women), based on ridits for education, income and occupation, Norway 2002-2019.

## Data Availability

The data are not publicly available due to privacy and ethical concerns, but may be obtained by researchers, free of charge, from Sikt – Norwegian Agency for Shared Services in Education and Research (https://sikt.no/en/about-sikt) after a Data Protection Impact Assessment (DPIA) has been completed.

## Abbreviations

AIC: The Akaike information criterion
BIC: The Bayesian information criterion
BMI: Body mass index
EHIS: European Health Interview Survey
GSRH: Good self-rated health
pp: Percentage points
RII: Relative Index of Inequality
SEP: Socioeconomic position
SII: Slope Index of Inequality
SRH: Self-rated health

## Footnotes

1 See https://ec.europa.eu/eurostat/web/microdata/european-health-interview-survey.

2 This pertained to 79%, 83%, 82%, and 99.8% in 2002, 2005, 2008 and 2012, respectively. The remaining interviews were conducted face-to-face. Use of face-to-face interviews was preferred for respondents above 67 years of age and respondents with 2 + children aged 6–15.

3 Defined by Statistic Norway as neither having been absent from work nor performed paid work during the last week.

4 Formally, in Stata the dydx-command calculates the probability point change in y with one unit change in x, while the eydx-command calculates the percent change in ln(y) with one unit change in x.

5 Goodman and Kruskal’s gamma coefficient was 0.60 for education-occupation, 0.33 for education-income and 0.38 for occupation-income.

6 According to Eurostat and OECD, Norway’s gini coefficient was around 0.05 points below that of the EU between 2002 and 2019, see https://ec.europa.eu/eurostat/databrowser/product/view/ilc_di12 and https://www.oecd.org/social/soc/49499779.pdf.
