# Supplementary material for: Changes over time in social inequality in adult self-rated health: the case of Norway 2002–2019
Source: BMC Public Health. 2025 Nov 11;25:3894. doi: 10.1186/s12889-025-25248-w (PMC12606821; doi:10.1186/s12889-025-25248-w)
Supplement: Supplementary file 1 — Additional file 1. Sample size, response rates and descriptive changes over time for the pooled sample. Table A1a. Sample size and response rates in interview surveys of living conditions 2002-2019. Table A1b. Distributions of variables in Model 4, percent (%) and number of respondents (n), 2002-2019, men and women combined. [file 12889_2025_25248_MOESM1_ESM.docx]

# Additional file 1. Sample size, response rates and descriptive changes over time for the pooled sample

**Table A1a. Sample size and response rates in interview surveys of living conditions 2002-2019**

|  | **2002** | **2005** | **2008** | **2012** | **2015 (EHIS)** | **2019 (EHIS)** |
| --- | --- | --- | --- | --- | --- | --- |
| Overall sample (n) | 10000 | 10000 | 10000 | 10000 | 14000 | 14000 |
| No contact ^a^ (n) | 302 | 303 | 316 | 229 | 252 | 181 |
| Gross sample (n) | 9698 | 9697 | 9684 | 9771 | 13748 | 13819 |
| Nonresponse (n) | 2871 | 2931 | 3219 | 4111 | 5584 | 5906 |
| Net sample (n) | 6827 | 6766 | 6465 | 5660 | 8164 | 7913 |
| Response rate^b^ (%) | 70.4 | 69.8 | 66.8 | 57.9 | 59.4 | 57.3 |
| Telephone interviews^c^ (%) | 78.6 | 83.0 | 82.0 | 99.8 | 100.0 | 100.0 |
| Study sample^d^ (n) | 4913 | 4853 | 4612 | 3864 | 5483 | 5287 |

^a^ Dead, moved to another country or in institution.

^b^ Net sample/gross sample.

^c^ In 2002-2008, use of face-to-face interviews was preferred for respondents aged 67 years or more (not included in our sample), and respondents with two or more children aged 6-15. For the year 2005, it is not entirely clear from the survey documentation if this percentage represents the whole interview survey or a sub sample.

^d^ The study sample consists of respondents aged 25 to 66 years.

**Table A1b. Distributions of variables in Model 4, percent (%) and number of respondents (n), 2002-2019, men and women combined**^a^

|  |  | **2002** | | **2005** | | **2008** | | **2012** | | **2015** | | **2019** | | **All years** | |
| --- | --- | --- | --- | --- | --- | --- | --- | --- | --- | --- | --- | --- | --- | --- | --- |
|  |  | **%** | **n** | **%** | **n** | **%** | **n** | **%** | **n** | **%** | **n** | **%** | **n** | **%** | **n** |
| **Good self-reported health** | Yes | 82.4 | 4911 | 82.1 | 4851 | 81.6 | 4602 | 78.2 | 3859 | 80.9 | 5480 | 80.1 | 5286 | 81.0 | 28989 |
|  | No | 17.6 | 4911 | 17.9 | 4851 | 18.4 | 4602 | 21.8 | 3859 | 19.1 | 5480 | 19.9 | 5286 | 19.0 | 28989 |
| **Age** | Mean (not percent) | 44.2 | 4913 | 44.7 | 4853 | 45.6 | 4612 | 46.4 | 3864 | 46.1 | 5483 | 46.1 | 5287 | 45.5 | 29012 |
| **Sex** | Men | 51.0 | 4913 | 50.2 | 4853 | 49.5 | 4612 | 50.8 | 3864 | 50.3 | 5483 | 49.9 | 5287 | 50.3 | 29012 |
|  | Women | 49.0 | 4913 | 49.8 | 4853 | 50.5 | 4612 | 49.2 | 3864 | 49.7 | 5483 | 50.1 | 5287 | 49.7 | 29012 |
| **Marital status** | Married | 58.4 | 4913 | 54.6 | 4853 | 55.6 | 4606 | 56.0 | 3864 | 52.6 | 5481 | 50.0 | 5283 | 54.4 | 29000 |
|  | Cohabiting | 17.9 | 4913 | 19.7 | 4853 | 19.5 | 4606 | 18.4 | 3864 | 18.9 | 5481 | 20.3 | 5283 | 19.1 | 29000 |
|  | No partner | 23.7 | 4913 | 25.7 | 4853 | 24.9 | 4606 | 25.6 | 3864 | 28.5 | 5481 | 29.7 | 5283 | 26.5 | 29000 |
| **Rural/urban area** | >100 000 | 22.3 | 4897 | 30.4 | 3845 | 28.6 | 3688 | 29.9 | 3003 | 27.7 | 5483 | 26.3 | 5274 | 27.2 | 26190 |
|  | 20 000-100 000 | 19.8 | 4897 | 24.9 | 3845 | 28.6 | 3688 | 27.1 | 3003 | 15.7 | 5483 | 18.5 | 5274 | 21.5 | 26190 |
|  | 2 000-19 999 | 25.7 | 4897 | 33.8 | 3845 | 32.5 | 3688 | 33.0 | 3003 | 24.2 | 5483 | 23.4 | 5274 | 27.9 | 26190 |
|  | <2 000 | 32.2 | 4897 | 10.9 | 3845 | 10.4 | 3688 | 10.0 | 3003 | 32.4 | 5483 | 31.8 | 5274 | 23.4 | 26190 |
| **Adjusted household income quintiles** | q5 (highest) | 20.5 | 4806 | 20.5 | 4714 | 20.5 | 4525 | 19.6 | 3772 | 20.7 | 5343 | 20.6 | 5157 | 20.4 | 28317 |
|  | q4 | 20.8 | 4806 | 20.5 | 4714 | 20.5 | 4525 | 19.8 | 3772 | 20.0 | 5343 | 20.2 | 5157 | 20.3 | 28317 |
|  | q3 | 20.5 | 4806 | 20.3 | 4714 | 20.2 | 4525 | 20.9 | 3772 | 20.2 | 5343 | 20.1 | 5157 | 20.3 | 28317 |
|  | q2 | 19.4 | 4806 | 19.4 | 4714 | 19.0 | 4525 | 19.3 | 3772 | 19.2 | 5343 | 19.7 | 5157 | 19.4 | 28317 |
|  | q1 (lowest) | 18.8 | 4806 | 19.3 | 4714 | 19.9 | 4525 | 20.4 | 3772 | 19.8 | 5343 | 19.5 | 5157 | 19.6 | 28317 |
| **Education** | Long | 31.7 | 4840 | 33.1 | 4746 | 37.3 | 4421 | 42.3 | 3802 | 43.8 | 5348 | 46.3 | 5129 | 39.2 | 28286 |
|  | Medium | 55.9 | 4840 | 57.0 | 4746 | 44.7 | 4421 | 43.1 | 3802 | 41.0 | 5348 | 38.3 | 5129 | 46.6 | 28286 |
|  | Short | 12.4 | 4840 | 9.8 | 4746 | 18.0 | 4421 | 14.5 | 3802 | 15.2 | 5348 | 15.4 | 5129 | 14.2 | 28286 |
| **Occupation/ employment** | High-skilled white collar | 46.0 | 4909 | 39.5 | 4852 | 44.9 | 4605 | 49.4 | 3859 | 48.8 | 5461 | 50.0 | 5267 | 46.4 | 28953 |
|  | Low-skilled white collar | 18.2 | 4909 | 21.1 | 4852 | 20.4 | 4605 | 18.0 | 3859 | 19.2 | 5461 | 18.5 | 5267 | 19.3 | 28953 |
|  | High-skilled blue collar | 9.6 | 4909 | 12.4 | 4852 | 10.7 | 4605 | 10.0 | 3859 | 8.0 | 5461 | 8.4 | 5267 | 9.8 | 28953 |
|  | Low-skilled blue collar | 8.9 | 4909 | 8.4 | 4852 | 7.4 | 4605 | 6.4 | 3859 | 7.0 | 5461 | 7.1 | 5267 | 7.5 | 28953 |
|  | Not employed | 17.3 | 4909 | 18.5 | 4852 | 16.7 | 4605 | 16.2 | 3859 | 17.1 | 5461 | 16.0 | 5267 | 17.0 | 28953 |
| **Smoking status** | Do not smoke | 59.1 | 4908 | 62.9 | 4851 | 67.5 | 4596 | 72.2 | 3852 | 77.8 | 5468 | 80.8 | 5272 | 70.3 | 28947 |
|  | Occasionally | 11.1 | 4908 | 10.1 | 4851 | 9.4 | 4596 | 9.0 | 3852 | 7.1 | 5468 | 7.7 | 5272 | 9.0 | 28947 |
|  | Daily | 29.8 | 4908 | 27.0 | 4851 | 23.1 | 4596 | 18.7 | 3852 | 15.1 | 5468 | 11.5 | 5272 | 20.7 | 28947 |
| **Exercise** | ≥ 1 time/week | 64.0 | 4906 | 71.0 | 4852 | 73.7 | 4598 | 80.7 | 3848 | 73.0 | 5463 | 73.8 | 5273 | 72.4 | 28940 |
|  | < 1 time/week | 11.9 | 4906 | 14.2 | 4852 | 14.4 | 4598 | 10.1 | 3848 | 13.9 | 5463 | 13.6 | 5273 | 13.1 | 28940 |
|  | Never | 24.2 | 4906 | 14.9 | 4852 | 11.9 | 4598 | 9.2 | 3848 | 13.1 | 5463 | 12.6 | 5273 | 14.5 | 28940 |
| **Body Mass Index** | Normal/underweight | 54.0 | 4835 | 52.1 | 4782 | 50.7 | 4518 | 50.1 | 3819 | 46.9 | 5390 | 45.0 | 5181 | 49.7 | 28525 |
|  | Overweight | 36.9 | 4835 | 37.9 | 4782 | 37.9 | 4518 | 39.0 | 3819 | 38.6 | 5390 | 39.2 | 5181 | 38.2 | 28525 |
|  | Obesity | 9.1 | 4835 | 10.0 | 4782 | 11.4 | 4518 | 10.9 | 3819 | 14.5 | 5390 | 15.8 | 5181 | 12.1 | 28525 |
| **People to ask in case of personal trouble?** | 3+ | 70.5 | 4871 | 79.1 | 4841 | 79.5 | 4581 | 83.5 | 3823 | 81.0 | 5450 | 83.5 | 5254 | 79.5 | 28820 |
|  | 1 or 2 | 28.2 | 4871 | 19.4 | 4841 | 19.2 | 4581 | 15.2 | 3823 | 17.6 | 5450 | 15.6 | 5254 | 19.3 | 28820 |
|  | None | 1.3 | 4871 | 1.5 | 4841 | 1.3 | 4581 | 1.2 | 3823 | 1.3 | 5450 | 1.0 | 5254 | 1.3 | 28820 |
| **Someone to confide in?** | Yes | 98.2 | 4902 | 97.3 | 4851 | 95.2 | 4593 | 95.6 | 3843 | 97.0 | 5457 | 97.2 | 5268 | 96.8 | 28914 |
|  | No | 1.8 | 4902 | 2.7 | 4851 | 4.8 | 4593 | 4.4 | 3843 | 3.0 | 5457 | 2.8 | 5268 | 3.2 | 28914 |

^a^ Distributions by geographic region are not shown (available on request)
