# Supplementary material for: Changes over time in social inequality in adult self-rated health: the case of Norway 2002–2019
Source: BMC Public Health. 2025 Nov 11;25:3894. doi: 10.1186/s12889-025-25248-w (PMC12606821; doi:10.1186/s12889-025-25248-w)
Supplement: Supplementary file 2 — Additional file 2. Model choice. Table A2a. Odds ratios (OR), standard errors (SE) and p-values (p) from Model 1 to Model 4, men. Table A2b. Odds ratios (OR), standard errors (SE) and p-values (p) from Model 1 to Model 4, women. Figure A2a. Probability of good self-rated health among men and women, Norway 2002-2019. Estimates from all models. [file 12889_2025_25248_MOESM2_ESM.docx]

# Additional file 2. Model choice

Table A2 shows the odds ratios from the four models for men (a) and women (b), respectively. Both BIC and AIC strongly suggested that Model 4 fitted the data best.

Figure A2a shows that the probabilities of good self-rated health (GSRH) varied little between years, regardless of statistical model. There was, however, a dip in GSRH among men in 2012 compared to adjacent years, and this was consistent across models.

| **Table A2a. Odds ratios (OR), standard errors (SE) and p-values (p) from Model 1 to Model 4, men** | | | | | | | | | | | | | |
| --- | --- | --- | --- | --- | --- | --- | --- | --- | --- | --- | --- | --- | --- |
|  |  | **Model 1** | | | **Model 2** | | | **Model 3** | | | **Model 4** | | |
|  |  | **OR** | **SE** | **p-value** | **OR** | **SE** | **p-value** | **OR** | **SE** | **p-value** | **OR** | **SE** | **p-value** |
| **Year** | 2002 | 1.000 | . | . | 1.000 | . | . | 1.000 | . | . | 1.000 | . | . |
|  | 2005 | 0.947 | 0.080 | 0.515 | 0.934 | 0.085 | 0.450 | 0.907 | 0.084 | 0.290 | 0.894 | 0.083 | 0.230 |
|  | 2008 | 1.100 | 0.097 | 0.278 | 1.045 | 0.099 | 0.644 | 0.994 | 0.097 | 0.954 | 0.994 | 0.098 | 0.951 |
|  | 2012 | 0.740 | 0.063 | 0.000 | 0.664 | 0.061 | 0.000 | 0.615 | 0.059 | 0.000 | 0.609 | 0.059 | 0.000 |
|  | 2015 | 0.990 | 0.074 | 0.889 | 0.936 | 0.076 | 0.411 | 0.919 | 0.077 | 0.312 | 0.898 | 0.076 | 0.205 |
|  | 2019 | 0.977 | 0.074 | 0.760 | 0.860 | 0.070 | 0.064 | 0.843 | 0.071 | 0.043 | 0.815 | 0.070 | 0.017 |
|  |  |  |  |  |  |  |  |  |  |  |  |  |  |
| **Age** | Age (linear) | 0.963 | 0.002 | 0.000 | 0.977 | 0.002 | 0.000 | 0.977 | 0.002 | 0.000 | 0.978 | 0.002 | 0.000 |
|  |  |  |  |  |  |  |  |  |  |  |  |  |  |
| **Civil status** | Married | 1.000 | . | . | 1.000 | . | . | 1.000 | . | . | 1.000 | . | . |
|  | Cohabiting | 0.855 | 0.058 | 0.022 | 0.914 | 0.066 | 0.212 | 0.924 | 0.068 | 0.280 | 0.929 | 0.068 | 0.317 |
|  | No partner | 0.530 | 0.029 | 0.000 | 0.781 | 0.049 | 0.000 | 0.806 | 0.052 | 0.001 | 0.820 | 0.053 | 0.002 |
|  |  |  |  |  |  |  |  |  |  |  |  |  |  |
| **Rural/urban area** | >100 000 | 1.000 | . | . | 1.000 | . | . | 1.000 | . | . | 1.000 | . | . |
|  | 20 000-100 000 | 0.980 | 0.079 | 0.801 | 1.028 | 0.080 | 0.722 | 1.038 | 0.082 | 0.641 | 1.036 | 0.083 | 0.661 |
|  | 2 000-19 999 | 0.868 | 0.064 | 0.054 | 0.912 | 0.066 | 0.204 | 0.931 | 0.069 | 0.336 | 0.936 | 0.070 | 0.376 |
|  | <2 000 | 0.723 | 0.055 | 0.000 | 0.815 | 0.061 | 0.006 | 0.845 | 0.065 | 0.029 | 0.848 | 0.066 | 0.034 |
|  |  |  |  |  |  |  |  |  |  |  |  |  |  |
| **Geographic region** | Akershus & Oslo | 1.000 | . | . |  |  |  |  |  |  |  |  |  |
|  | Hedmark & Oppland | 0.964 | 0.103 | 0.729 |  |  |  |  |  |  |  |  |  |
|  | Other Eastern Norway | 0.808 | 0.068 | 0.012 |  |  |  |  |  |  |  |  |  |
|  | Agder & Rogaland | 0.910 | 0.079 | 0.280 |  |  |  |  |  |  |  |  |  |
|  | Western Norway | 0.989 | 0.085 | 0.901 |  |  |  |  |  |  |  |  |  |
|  | Trønderlag | 0.993 | 0.099 | 0.942 |  |  |  |  |  |  |  |  |  |
|  | Northern Norway | 0.838 | 0.078 | 0.058 |  |  |  |  |  |  |  |  |  |
|  |  |  |  |  |  |  |  |  |  |  |  |  |  |
| **Adjusted household income quintiles** | q5 (highest) |  |  |  | 1.000 | . | . | 1.000 | . | . | 1.000 | . | . |
|  | q4 |  |  |  | 0.827 | 0.075 | 0.037 | 0.849 | 0.078 | 0.077 | 0.849 | 0.079 | 0.077 |
|  | q3 |  |  |  | 0.672 | 0.059 | 0.000 | 0.688 | 0.062 | 0.000 | 0.691 | 0.062 | 0.000 |
|  | q2 |  |  |  | 0.627 | 0.056 | 0.000 | 0.656 | 0.060 | 0.000 | 0.658 | 0.060 | 0.000 |
|  | q1 (lowest) |  |  |  | 0.503 | 0.045 | 0.000 | 0.533 | 0.049 | 0.000 | 0.544 | 0.051 | 0.000 |
|  |  |  |  |  |  |  |  |  |  |  |  |  |  |
| **Education** | Long |  |  |  | 1.000 | . | . | 1.000 | . | . | 1.000 | . | . |
|  | Medium |  |  |  | 0.731 | 0.048 | 0.000 | 0.841 | 0.057 | 0.011 | 0.846 | 0.058 | 0.014 |
|  | Short |  |  |  | 0.598 | 0.051 | 0.000 | 0.736 | 0.065 | 0.000 | 0.751 | 0.066 | 0.001 |
|  |  |  |  |  |  |  |  |  |  |  |  |  |  |
| **Occupation/ Employment** | High-skilled white collar |  |  |  | 1.000 | . | . | 1.000 | . | . | 1.000 | . | . |
|  | Low-skilled white collar |  |  |  | 0.792 | 0.070 | 0.008 | 0.806 | 0.073 | 0.017 | 0.804 | 0.073 | 0.016 |
|  | High-skilled blue collar |  |  |  | 0.900 | 0.074 | 0.199 | 0.944 | 0.079 | 0.489 | 0.944 | 0.079 | 0.491 |
|  | Low-skilled blue collar |  |  |  | 0.823 | 0.076 | 0.036 | 0.944 | 0.090 | 0.542 | 0.972 | 0.093 | 0.766 |
|  | Not employed |  |  |  | 0.224 | 0.017 | 0.000 | 0.230 | 0.018 | 0.000 | 0.236 | 0.018 | 0.000 |
|  |  |  |  |  |  |  |  |  |  |  |  |  |  |
| **Smoking status** | Do not smoke |  |  |  |  |  |  | 1.000 | . | . | 1.000 | . | . |
|  | Occasionally |  |  |  |  |  |  | 1.056 | 0.102 | 0.574 | 1.039 | 0.101 | 0.691 |
|  | Daily |  |  |  |  |  |  | 0.687 | 0.044 | 0.000 | 0.679 | 0.044 | 0.000 |
|  |  |  |  |  |  |  |  |  |  |  |  |  |  |
| **Exercise** | ≥ 1 time/week |  |  |  |  |  |  | 1.000 | . | . | 1.000 | . | . |
|  | < 1 time/week |  |  |  |  |  |  | 0.692 | 0.049 | 0.000 | 0.707 | 0.051 | 0.000 |
|  | Never |  |  |  |  |  |  | 0.568 | 0.038 | 0.000 | 0.590 | 0.040 | 0.000 |
|  |  |  |  |  |  |  |  |  |  |  |  |  |  |
| **Body Mass Index** | Normal/underweight |  |  |  |  |  |  | 1.000 | . | . | 1.000 | . | . |
|  | Overweight |  |  |  |  |  |  | 0.765 | 0.045 | 0.000 | 0.769 | 0.046 | 0.000 |
|  | Obesity |  |  |  |  |  |  | 0.341 | 0.025 | 0.000 | 0.342 | 0.026 | 0.000 |
|  |  |  |  |  |  |  |  |  |  |  |  |  |  |
| **People to ask in case of personal trouble?** | 3+ |  |  |  |  |  |  |  |  |  | 1.000 | . | . |
|  | 1 or 2 |  |  |  |  |  |  |  |  |  | 0.862 | 0.052 | 0.015 |
|  | No one |  |  |  |  |  |  |  |  |  | 0.821 | 0.165 | 0.328 |
|  |  |  |  |  |  |  |  |  |  |  |  |  |  |
| **Someone to confide in?** | Yes |  |  |  |  |  |  |  |  |  | 1.000 | . | . |
|  | No |  |  |  |  |  |  |  |  |  | 0.699 | 0.089 | 0.005 |
| **Constant** |  | 42.407 | 5.617 | 0.000 | 48.350 | 7.376 | 0.000 | 69.639 | 11.296 | 0.000 | 69.497 | 11.329 | 0.000 |
| **BIC** |  | 11922 |  |  | 10462 |  |  | 10091 |  |  | 10039 |  |  |
| **AIC** |  | 11787 |  |  | 10299 |  |  | 9883 |  |  | 9809 |  |  |
| **N** |  | 13116 |  |  | 12441 |  |  | 12367 |  |  | 12293 |  |  |

| **Table A2b. Odds ratios (OR), standard errors (SE) and p-values (p) from Model 1 to Model 4, women** | | | | | | | | | | | | | |
| --- | --- | --- | --- | --- | --- | --- | --- | --- | --- | --- | --- | --- | --- |
|  |  | **Model 1** | | | **Model 2** | | | **Model 3** | | | **Model 4** | | |
|  |  | **OR** | **SE** | **p-value** | **OR** | **SE** | **p-value** | **OR** | **SE** | **p-value** | **OR** | **SE** | **p-value** |
| **Year** | 2002 | 1.000 | . | . | 1.000 | . | . | 1.000 | . | . | 1.000 | . | . |
|  | 2005 | 1.120 | 0.092 | 0.168 | 1.053 | 0.094 | 0.561 | 1.053 | 0.099 | 0.583 | 1.041 | 0.098 | 0.668 |
|  | 2008 | 0.925 | 0.074 | 0.326 | 0.847 | 0.075 | 0.060 | 0.773 | 0.071 | 0.005 | 0.768 | 0.071 | 0.004 |
|  | 2012 | 0.986 | 0.085 | 0.868 | 0.810 | 0.076 | 0.025 | 0.708 | 0.070 | 0.000 | 0.698 | 0.069 | 0.000 |
|  | 2015 | 1.027 | 0.075 | 0.718 | 0.866 | 0.070 | 0.074 | 0.821 | 0.069 | 0.019 | 0.809 | 0.069 | 0.012 |
|  | 2019 | 0.950 | 0.069 | 0.478 | 0.732 | 0.059 | 0.000 | 0.685 | 0.058 | 0.000 | 0.669 | 0.057 | 0.000 |
|  |  |  |  |  |  |  |  |  |  |  |  |  |  |
| **Age** | Age (linear) | 0.966 | 0.002 | 0.000 | 0.983 | 0.002 | 0.000 | 0.984 | 0.002 | 0.000 | 0.984 | 0.002 | 0.000 |
|  |  |  | . | . |  |  |  |  |  |  |  |  |  |
| **Civil status** | Married | 1.000 | . | . | 1.000 | . | . | 1.000 | . | . | 1.000 | . | . |
|  | Cohabiting | 0.948 | 0.064 | 0.432 | 1.022 | 0.074 | 0.763 | 1.071 | 0.080 | 0.361 | 1.067 | 0.080 | 0.386 |
|  | No partner | 0.642 | 0.033 | 0.000 | 0.880 | 0.056 | 0.044 | 0.905 | 0.060 | 0.134 | 0.917 | 0.061 | 0.193 |
|  |  |  |  |  |  |  |  |  |  |  |  |  |  |
| **Rural/urban area** | >100 000 | 1.000 | . | . | 1.000 | . | . | 1.000 | . | . | 1.000 | . | . |
|  | 20 000-100 000 | 0.888 | 0.067 | 0.118 | 0.961 | 0.071 | 0.591 | 0.987 | 0.075 | 0.862 | 0.991 | 0.075 | 0.902 |
|  | 2 000-19 999 | 0.837 | 0.058 | 0.010 | 0.948 | 0.065 | 0.433 | 0.990 | 0.070 | 0.884 | 0.989 | 0.070 | 0.871 |
|  | <2 000 | 0.781 | 0.058 | 0.001 | 0.882 | 0.064 | 0.085 | 0.950 | 0.072 | 0.501 | 0.952 | 0.072 | 0.516 |
|  |  |  |  |  |  |  |  |  |  |  |  |  |  |
| **Geographic region** | Akershus & Oslo | 1.000 | . | . |  |  |  |  |  |  |  |  |  |
|  | Hedmark & Oppland | 0.911 | 0.092 | 0.355 |  |  |  |  |  |  |  |  |  |
|  | Other Eastern Norway | 0.881 | 0.070 | 0.108 |  |  |  |  |  |  |  |  |  |
|  | Agder & Rogaland | 0.855 | 0.070 | 0.056 |  |  |  |  |  |  |  |  |  |
|  | Western Norway | 0.999 | 0.081 | 0.993 |  |  |  |  |  |  |  |  |  |
|  | Trønderlag | 0.939 | 0.090 | 0.510 |  |  |  |  |  |  |  |  |  |
|  | Northern Norway | 0.904 | 0.082 | 0.264 |  |  |  |  |  |  |  |  |  |
|  |  |  |  |  |  |  |  |  |  |  |  |  |  |
| **Adjusted household income quintiles** | q5 (highest) |  |  |  | 1.000 | . | . | 1.000 | . | . | 1.000 | . | . |
|  | q4 |  |  |  | 0.641 | 0.057 | 0.000 | 0.661 | 0.060 | 0.000 | 0.667 | 0.061 | 0.000 |
|  | q3 |  |  |  | 0.564 | 0.050 | 0.000 | 0.585 | 0.053 | 0.000 | 0.590 | 0.053 | 0.000 |
|  | q2 |  |  |  | 0.544 | 0.049 | 0.000 | 0.610 | 0.056 | 0.000 | 0.612 | 0.057 | 0.000 |
|  | q1 (lowest) |  |  |  | 0.457 | 0.043 | 0.000 | 0.523 | 0.051 | 0.000 | 0.534 | 0.053 | 0.000 |
|  |  |  |  |  |  |  |  |  |  |  |  |  |  |
| **Education** | Long |  |  |  | 1.000 | . | . | 1.000 | . | . | 1.000 | . | . |
|  | Medium |  |  |  | 0.708 | 0.046 | 0.000 | 0.760 | 0.051 | 0.000 | 0.770 | 0.052 | 0.000 |
|  | Short |  |  |  | 0.537 | 0.043 | 0.000 | 0.619 | 0.052 | 0.000 | 0.636 | 0.054 | 0.000 |
|  |  |  |  |  |  |  |  |  |  |  |  |  |  |
| **Occupation/ Employment** | High-skilled white collar |  |  |  | 1.000 | . | . | 1.000 | . | . | 1.000 | . | . |
|  | Low-skilled white collar |  |  |  | 0.758 | 0.056 | 0.000 | 0.785 | 0.059 | 0.001 | 0.789 | 0.060 | 0.002 |
|  | High-skilled blue collar |  |  |  | 0.775 | 0.143 | 0.167 | 0.764 | 0.143 | 0.150 | 0.765 | 0.144 | 0.154 |
|  | Low-skilled blue collar |  |  |  | 0.762 | 0.098 | 0.035 | 0.806 | 0.107 | 0.104 | 0.806 | 0.107 | 0.105 |
|  | Not employed |  |  |  | 0.177 | 0.012 | 0.000 | 0.183 | 0.013 | 0.000 | 0.189 | 0.014 | 0.000 |
|  |  |  |  |  |  |  |  |  |  |  |  |  |  |
| **Smoking status** | Do not smoke |  |  |  |  |  |  | 1.000 | . | . | 1.000 | . | . |
|  | Occasionally |  |  |  |  |  |  | 1.012 | 0.099 | 0.907 | 1.009 | 0.100 | 0.929 |
|  | Daily |  |  |  |  |  |  | 0.642 | 0.040 | 0.000 | 0.645 | 0.041 | 0.000 |
|  |  |  |  |  |  |  |  |  |  |  |  |  |  |
| **Exercise** | ≥ 1 time/week |  |  |  |  |  |  | 1.000 | . | . | 1.000 | . | . |
|  | < 1 time/week |  |  |  |  |  |  | 0.801 | 0.063 | 0.005 | 0.813 | 0.064 | 0.009 |
|  | Never |  |  |  |  |  |  | 0.593 | 0.043 | 0.000 | 0.616 | 0.046 | 0.000 |
|  |  |  |  |  |  |  |  |  |  |  |  |  |  |
| **Body Mass Index** | Normal/underweight |  |  |  |  |  |  | 1.000 | . | . | 1.000 | . | . |
|  | Overweight |  |  |  |  |  |  | 0.709 | 0.041 | 0.000 | 0.709 | 0.041 | 0.000 |
|  | Obesity |  |  |  |  |  |  | 0.364 | 0.027 | 0.000 | 0.362 | 0.027 | 0.000 |
|  |  |  |  |  |  |  |  |  |  |  |  |  |  |
| **People to ask in case of personal trouble?** | 3+ |  |  |  |  |  |  |  |  |  | 1.000 | . | . |
|  | 1 or 2 |  |  |  |  |  |  |  |  |  | 0.790 | 0.052 | 0.000 |
|  | No one |  |  |  |  |  |  |  |  |  | 0.678 | 0.168 | 0.118 |
|  |  |  |  |  |  |  |  |  |  |  |  |  |  |
| **Someone to confide in)** | Yes |  |  |  |  |  |  |  |  |  | 1.000 | . | . |
|  | No |  |  |  |  |  |  |  |  |  | 0.715 | 0.123 | 0.051 |
| **Constant** |  | 29.909 | 3.694 | 0.000 | 42.909 | 6.199 | 0.000 | 55.967 | 8.543 | 0.000 | 55.985 | 8.573 | 0.000 |
| **BIC** |  | 12641 |  |  | 10774 |  |  | 10211 |  |  | 10200 |  |  |
| **AIC** |  | 12507 |  |  | 10610 |  |  | 10003 |  |  | 9971 |  |  |
| **N** |  | 13056 |  |  | 12468 |  |  | 12159 |  |  | 12130 |  |  |

**Figure A2a. Probability of good self-reported health among men and women, Norway 2002-2019. Estimates from all models**

**
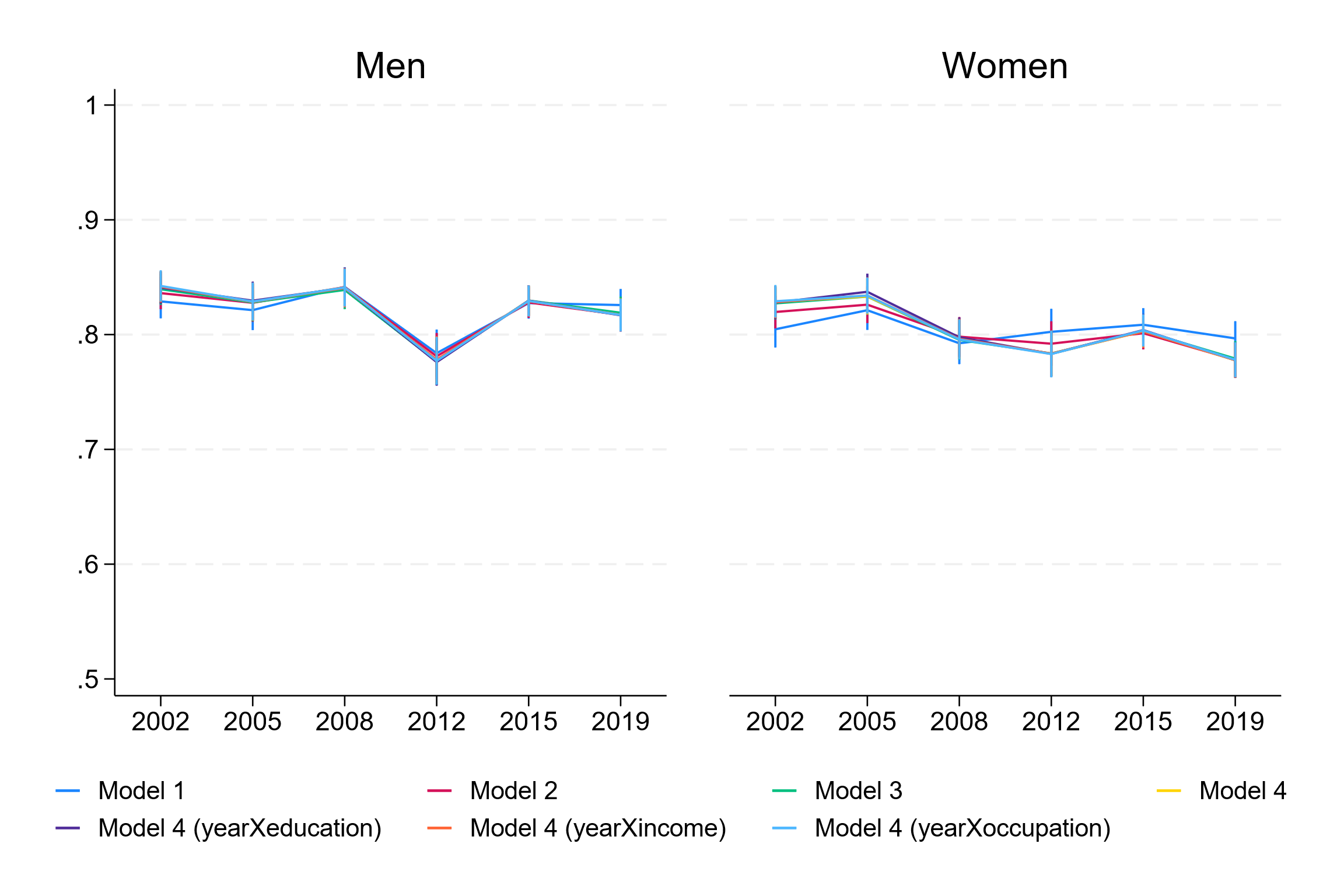
**
