# Supplementary material for: Changes over time in social inequality in adult self-rated health: the case of Norway 2002–2019
Source: BMC Public Health. 2025 Nov 11;25:3894. doi: 10.1186/s12889-025-25248-w (PMC12606821; doi:10.1186/s12889-025-25248-w)
Supplement: Supplementary file 3 — Additional file 3. Differences over time in percentage points and percentages. Figure A3a. Absolute (percentage points) differences (dydx) in reports of good health among men and women, by education, income and occupation, Norway 2002-2019. Figure A3b. Relative (percent) differences (eydx) in reports of good health among men and women, by education, income and occupation, Norway 2002-2019. Table A3a. Marginal mean probabilities (Margin), marginal effects (dydx), semi-elasticities (eydx), standard errors (se) and p-values (p) from Model 4 for men and women. [file 12889_2025_25248_MOESM3_ESM.docx]

# Additional file 3: Differences over time in percentage points and percentages

Additional file 3 shows absolute differences between groups over time in percentage points (Figure A3a) and relative differences in percent^[[1]](#footnote-1)^ (Figure A3b). The dashed line represents the base level. Table A3a reports the underlying estimates (from *margins*, *dydx* and *eydx*).

**Figure A3a. Absolute (percentage points) differences (dydx)** **in reports of good health among men and women, by education, income and occupation, Norway 2002-2019**

**
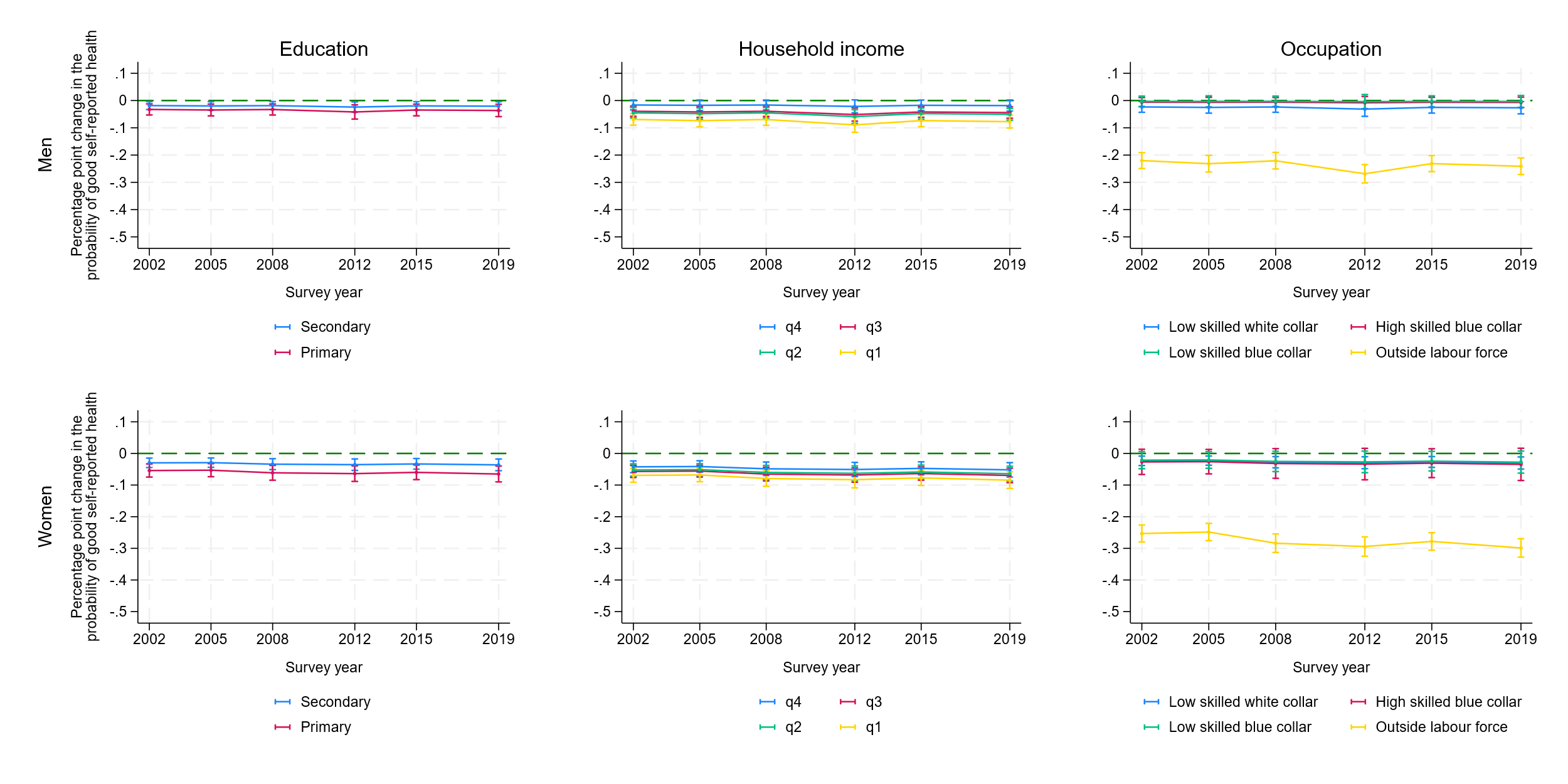
**

**Figure A3b. Relative (percent) differences (eydx) in reports of good health among men and women, by education, income and occupation, Norway 2002-2019**

**
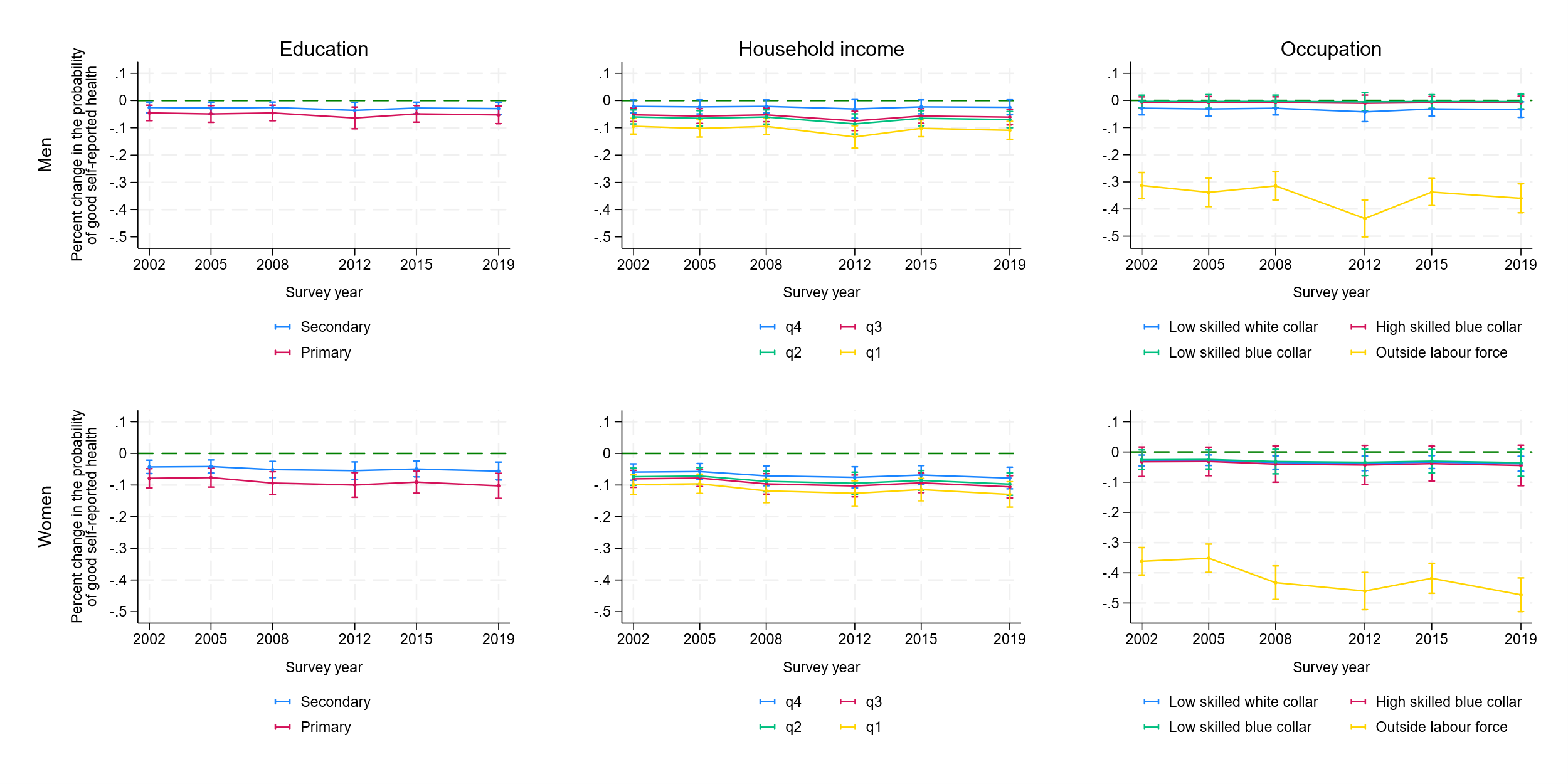
**

| **Table A3a. Marginal mean probabilities (Margin), marginal effects (dydx), semi-elasticities (eydx), standard errors (se) and p-values (p) from Model 4 for men and women**^a^ | | | | | | | | | | | | | | | | | | | |
| --- | --- | --- | --- | --- | --- | --- | --- | --- | --- | --- | --- | --- | --- | --- | --- | --- | --- | --- | --- |
|  |  | **Men** | | | | | | | | | **Women** | | | | | | | | |
|  |  | **Margin** | **SE** | **p** | **dydx** | **SE** | **p** | **eydx**^b^ | **SE** | **p** | **Margin** | **SE** | **p** | **dydx** | **SE** | **p** | **eydx**^b^ | **SE** | **p** |
| **Year** | 2002 | 0.842 | 0.007 | 0.000 | 0.000 |  |  | 0.000 |  |  | 0.829 | 0.007 | 0.000 | 0.000 |  |  | 0.000 |  |  |
|  | 2005 | 0.829 | 0.008 | 0.000 | -0.013 | 0.011 | 0.232 | -0.018 | 0.015 | 0.233 | 0.833 | 0.008 | 0.000 | 0.005 | 0.011 | 0.667 | 0.007 | 0.016 | 0.667 |
|  | 2008 | 0.841 | 0.009 | 0.000 | -0.001 | 0.011 | 0.951 | -0.001 | 0.016 | 0.951 | 0.796 | 0.009 | 0.000 | -0.032 | 0.011 | 0.005 | -0.049 | 0.018 | 0.005 |
|  | 2012 | 0.778 | 0.010 | 0.000 | -0.064 | 0.013 | 0.000 | -0.094 | 0.019 | 0.000 | 0.783 | 0.010 | 0.000 | -0.045 | 0.013 | 0.000 | -0.070 | 0.020 | 0.000 |
|  | 2015 | 0.829 | 0.007 | 0.000 | -0.012 | 0.010 | 0.204 | -0.018 | 0.014 | 0.204 | 0.803 | 0.007 | 0.000 | -0.026 | 0.010 | 0.012 | -0.039 | 0.016 | 0.012 |
|  |  |  |  |  |  |  |  |  |  |  |  |  |  |  |  |  |  |  |  |
| **Civil status** | Married | 0.833 | 0.004 | 0.000 | 0.000 |  |  | 0.000 |  |  |  |  |  | 0.000 |  |  | 0.000 |  |  |
|  | Cohabiting | 0.824 | 0.008 | 0.000 | -0.009 | 0.009 | 0.321 | -0.013 | 0.013 | 0.323 | 0.814 | 0.008 | 0.000 | 0.008 | 0.009 | 0.382 | 0.012 | 0.014 | 0.381 |
|  | No partner | 0.809 | 0.007 | 0.000 | -0.025 | 0.008 | 0.003 | -0.036 | 0.012 | 0.003 | 0.795 | 0.007 | 0.000 | -0.011 | 0.009 | 0.196 | -0.017 | 0.013 | 0.197 |
|  |  |  |  |  |  |  |  |  |  |  |  |  |  |  |  |  |  |  |  |
| **Rural/urban area** | >100 000 | 0.832 | 0.007 | 0.000 | 0.000 |  |  | 0.000 |  |  | 0.807 | 0.007 | 0.000 | 0.000 |  |  | 0.000 |  |  |
|  | 20 000-100 000 | 0.836 | 0.007 | 0.000 | 0.004 | 0.009 | 0.661 | 0.006 | 0.013 | 0.661 | 0.806 | 0.007 | 0.000 | -0.001 | 0.010 | 0.902 | -0.002 | 0.015 | 0.902 |
|  | 2 000-19 999 | 0.824 | 0.006 | 0.000 | -0.008 | 0.009 | 0.375 | -0.011 | 0.013 | 0.375 | 0.805 | 0.006 | 0.000 | -0.001 | 0.009 | 0.871 | -0.002 | 0.014 | 0.871 |
|  | <2 000 | 0.812 | 0.007 | 0.000 | -0.020 | 0.010 | 0.033 | -0.029 | 0.014 | 0.033 | 0.800 | 0.007 | 0.000 | -0.006 | 0.010 | 0.516 | -0.010 | 0.015 | 0.516 |
|  |  |  |  |  |  |  |  |  |  |  |  |  |  |  |  |  |  |  |  |
| **Adjusted household income quintiles** | q5 (highest) | 0.864 | 0.007 | 0.000 | 0.000 |  |  | 0.000 |  |  | 0.856 | 0.008 | 0.000 | 0.000 |  |  | 0.000 |  |  |
|  | q4 | 0.846 | 0.007 | 0.000 | -0.018 | 0.010 | 0.076 | -0.024 | 0.013 | 0.076 | 0.809 | 0.008 | 0.000 | -0.047 | 0.010 | 0.000 | -0.068 | 0.015 | 0.000 |
|  | q3 | 0.821 | 0.007 | 0.000 | -0.043 | 0.010 | 0.000 | -0.058 | 0.014 | 0.000 | 0.793 | 0.007 | 0.000 | -0.063 | 0.011 | 0.000 | -0.092 | 0.015 | 0.000 |
|  | q2 | 0.815 | 0.007 | 0.000 | -0.049 | 0.011 | 0.000 | -0.067 | 0.014 | 0.000 | 0.798 | 0.007 | 0.000 | -0.058 | 0.011 | 0.000 | -0.085 | 0.016 | 0.000 |
|  | q1 (lowest) | 0.789 | 0.008 | 0.000 | -0.075 | 0.011 | 0.000 | -0.104 | 0.016 | 0.000 | 0.779 | 0.008 | 0.000 | -0.077 | 0.012 | 0.000 | -0.113 | 0.018 | 0.000 |
|  |  |  |  |  |  |  |  |  |  |  |  |  |  |  |  |  |  |  |  |
| **Education** | Long | 0.842 | 0.006 | 0.000 | 0.000 |  |  | 0.000 |  |  | 0.830 | 0.006 | 0.000 | 0.000 |  |  | 0.000 |  |  |
|  | Medium | 0.822 | 0.004 | 0.000 | -0.020 | 0.008 | 0.013 | -0.028 | 0.011 | 0.012 | 0.797 | 0.005 | 0.000 | -0.033 | 0.008 | 0.000 | -0.049 | 0.012 | 0.000 |
|  |  |  |  |  |  |  |  |  |  |  |  |  |  |  |  |  |  |  |  |
|  | Short | 0.807 | 0.008 | 0.000 | -0.035 | 0.011 | 0.002 | -0.050 | 0.016 | 0.002 | 0.770 | 0.009 | 0.000 | -0.059 | 0.012 | 0.000 | -0.090 | 0.017 | 0.000 |
| **Occupation/ Employment** | High-skilled white collar | 0.865 | 0.005 | 0.000 | 0.000 |  |  | 0.000 |  |  | 0.873 | 0.005 | 0.000 | 0.000 |  |  | 0.000 |  |  |
|  | Low-skilled white collar | 0.840 | 0.009 | 0.000 | -0.026 | 0.011 | 0.020 | -0.032 | 0.014 | 0.021 | 0.846 | 0.006 | 0.000 | -0.027 | 0.009 | 0.002 | -0.033 | 0.011 | 0.002 |
|  | High-skilled blue collar | 0.859 | 0.008 | 0.000 | -0.006 | 0.009 | 0.494 | -0.008 | 0.012 | 0.495 | 0.842 | 0.022 | 0.000 | -0.031 | 0.023 | 0.185 | -0.038 | 0.029 | 0.194 |
|  | Low-skilled blue collar | 0.862 | 0.009 | 0.000 | -0.003 | 0.011 | 0.768 | -0.004 | 0.013 | 0.768 | 0.848 | 0.014 | 0.000 | -0.024 | 0.016 | 0.122 | -0.030 | 0.020 | 0.127 |
|  | Not employed | 0.631 | 0.013 | 0.000 | -0.234 | 0.014 | 0.000 | -0.345 | 0.023 | 0.000 | 0.597 | 0.011 | 0.000 | -0.276 | 0.013 | 0.000 | -0.415 | 0.021 | 0.000 |
|  |  |  |  |  |  |  |  |  |  |  |  |  |  |  |  |  |  |  |  |
| **Smoking** | Do not smoke | 0.836 | 0.004 | 0.000 | 0.000 |  |  | 0.000 |  |  | 0.818 | 0.004 | 0.000 | 0.000 |  |  | 0.000 |  |  |
|  | Occasionally | 0.841 | 0.011 | 0.000 | 0.004 | 0.011 | 0.689 | 0.006 | 0.016 | 0.688 | 0.819 | 0.011 | 0.000 | 0.001 | 0.012 | 0.929 | 0.002 | 0.018 | 0.929 |
|  | Daily | 0.786 | 0.008 | 0.000 | -0.050 | 0.009 | 0.000 | -0.073 | 0.013 | 0.000 | 0.758 | 0.008 | 0.000 | -0.059 | 0.009 | 0.000 | -0.092 | 0.014 | 0.000 |
|  |  |  |  |  |  |  |  |  |  |  |  |  |  |  |  |  |  |  |  |
| **Exercise** | ≥ 1 time/week | 0.844 | 0.004 | 0.000 | 0.000 |  |  | 0.000 |  |  | 0.817 | 0.004 | 0.000 | 0.000 |  |  | 0.000 |  |  |
|  | < 1 time/week | 0.801 | 0.009 | 0.000 | -0.043 | 0.009 | 0.000 | -0.061 | 0.014 | 0.000 | 0.790 | 0.010 | 0.000 | -0.027 | 0.011 | 0.011 | -0.041 | 0.016 | 0.012 |
|  | Never | 0.776 | 0.009 | 0.000 | -0.069 | 0.009 | 0.000 | -0.100 | 0.014 | 0.000 | 0.750 | 0.010 | 0.000 | -0.067 | 0.011 | 0.000 | -0.105 | 0.018 | 0.000 |
|  |  |  |  |  |  |  |  |  |  |  |  |  |  |  |  |  |  |  |  |
| **Body Mass Index** | Normal/underweight | 0.861 | 0.005 | 0.000 | 0.000 |  |  | 0.000 |  |  | 0.835 | 0.004 | 0.000 | 0.000 |  |  | 0.000 |  |  |
|  | Overweight | 0.831 | 0.005 | 0.000 | -0.030 | 0.007 | 0.000 | -0.040 | 0.009 | 0.000 | 0.792 | 0.006 | 0.000 | -0.043 | 0.007 | 0.000 | -0.064 | 0.011 | 0.000 |
|  | Obesity | 0.711 | 0.010 | 0.000 | -0.150 | 0.011 | 0.000 | -0.223 | 0.018 | 0.000 | 0.687 | 0.012 | 0.000 | -0.148 | 0.012 | 0.000 | -0.237 | 0.021 | 0.000 |
|  |  |  |  |  |  |  |  |  |  |  |  |  |  |  |  |  |  |  |  |
| **People to ask in case of personal trouble?** | 3+ | 0.830 | 0.004 | 0.000 | 0.000 |  |  | 0.000 |  |  | 0.811 | 0.004 | 0.000 | 0.000 |  |  | 0.000 |  |  |
|  | 1 or 2 | 0.812 | 0.007 | 0.000 | -0.018 | 0.008 | 0.017 | -0.027 | 0.011 | 0.018 | 0.780 | 0.008 | 0.000 | -0.031 | 0.009 | 0.001 | -0.048 | 0.014 | 0.001 |
|  | No one | 0.805 | 0.026 | 0.000 | -0.025 | 0.027 | 0.351 | -0.036 | 0.039 | 0.359 | 0.758 | 0.036 | 0.000 | -0.053 | 0.037 | 0.148 | -0.083 | 0.060 | 0.163 |
|  |  |  |  |  |  |  |  |  |  |  |  |  |  |  |  |  |  |  |  |
| **Someone to confide in?** | Yes | 0.827 | 0.003 | 0.000 | 0.000 |  |  | 0.000 |  |  | 0.806 | 0.003 | 0.000 | 0.000 |  |  | 0.000 |  |  |
|  | No | 0.780 | 0.018 | 0.000 | -0.047 | 0.018 | 0.009 | -0.070 | 0.028 | 0.012 | 0.760 | 0.025 | 0.000 | -0.046 | 0.025 | 0.069 | -0.073 | 0.041 | 0.078 |
| ^a^ In addition to the estimates shown, also age (linear) was included in the models.  ^b^ Formally, in Stata the dydx-command calculates the probability point change in y with one unit change in x, while the eydx-command calculates the percent change in ln(y) with one unit change in x. | | | | | | | | | | | | | | | | | | | |

1. Formally, in Stata the dydx-command calculates the probability point change in y with one unit change in x, while the eydx-command calculates the percent change in ln(y) with one unit change in x. For eydx-estimates <.10, the values for y and ln(y) will for all practical purposes be the same. [↑](#footnote-ref-1)
