# Supplementary material for: Changes over time in social inequality in adult self-rated health: the case of Norway 2002–2019
Source: BMC Public Health. 2025 Nov 11;25:3894. doi: 10.1186/s12889-025-25248-w (PMC12606821; doi:10.1186/s12889-025-25248-w)
Supplement: Supplementary file 4 — Additional file 4. Effect modification. Figure A4a. Probability of reporting good health among men and women, by education, income and occupation, Norway 2002-2019, from models including interaction terms between the relevant exposure variable and year. Figure A4b. Absolute (percentage point) difference (dydx) over time in reports of good health among men and women, by education, income and occupation, Norway 2002-2019, from models including interaction terms between the relevant exposure variable and year. Figure A4c. Relative (percent) difference (eydx) over time in reports of good health among men and women, by education, income and occupation, Norway 2002-2019, from models including interaction terms between the relevant exposure variable and year. Table A4a. Odds ratio (OR) estimates from 3 different models including an interaction term between year and the exposure variables. [file 12889_2025_25248_MOESM4_ESM.docx]

# Additional file 4: Effect modification

Figure A4a shows the variation across years reported as probabilities, whereas Figures A4b and A4c shows the absolute differences in percentage points (dydx, A4b) and relative differences in percentages (eydx, A4c) respectively^[[1]](#footnote-1)^. The dashed line represents the base level. We see that the patterns are largely similar in absolute and relative terms.

Table A4a presents the odds ratio (OR) estimates from three additional models including an interaction between year and each of the three exposure variables (Model 4e, Model 4i, Model 4o). None of the joint tests of the interactions were statistically significant at the 5% level.

**Figure A4a. Probability of reporting good self-reported health among men and women, by education, income and occupation, Norway 2002-2019, from models including interaction terms between the relevant exposure variable and year**

**
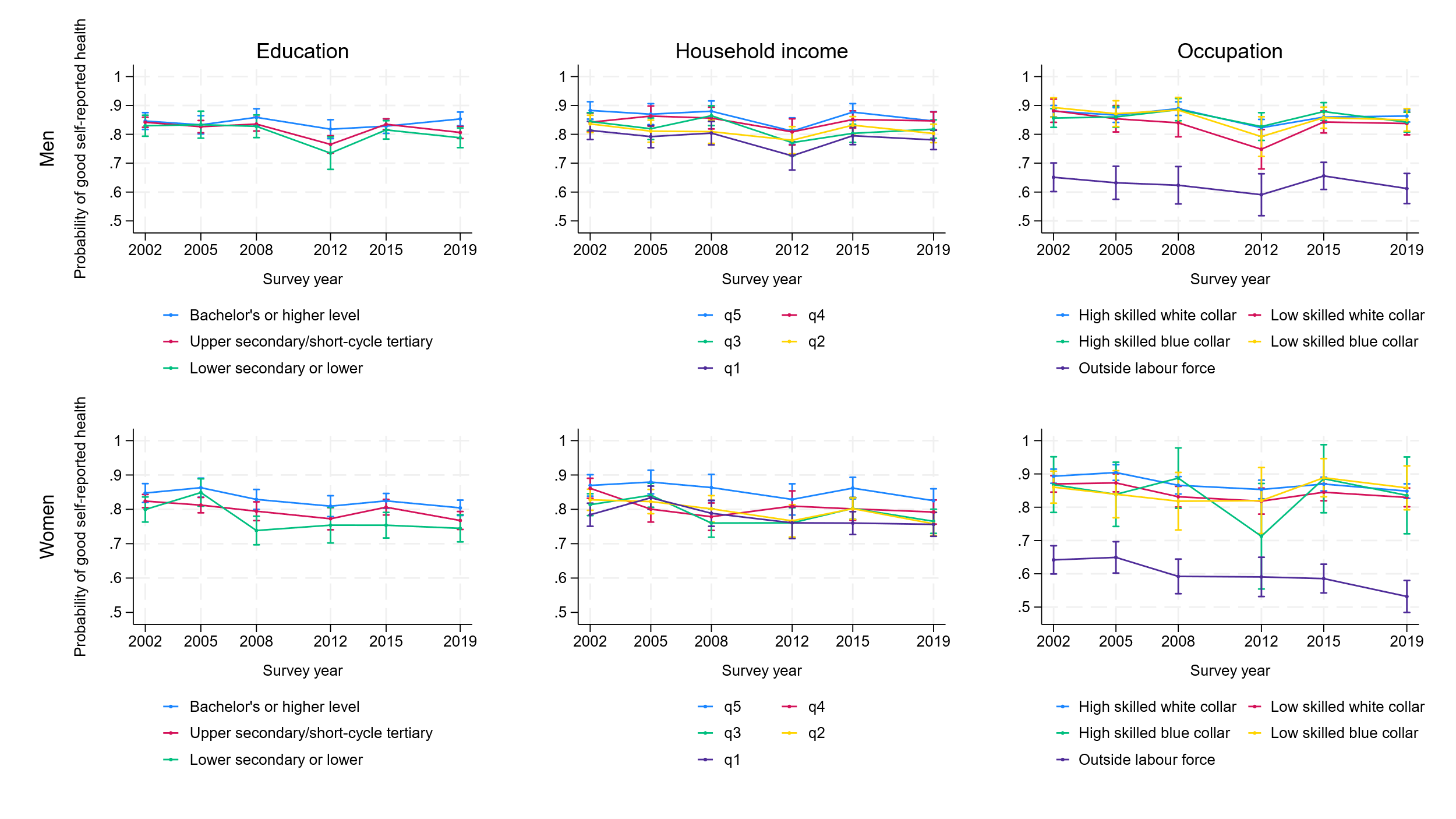
**

**Figure A4b. Absolute (percentage point) difference (dydx) over time in reports of good health among men and women, by education, income and occupation, Norway 2002-2019, from models including interaction terms between the relevant exposure variable and year**


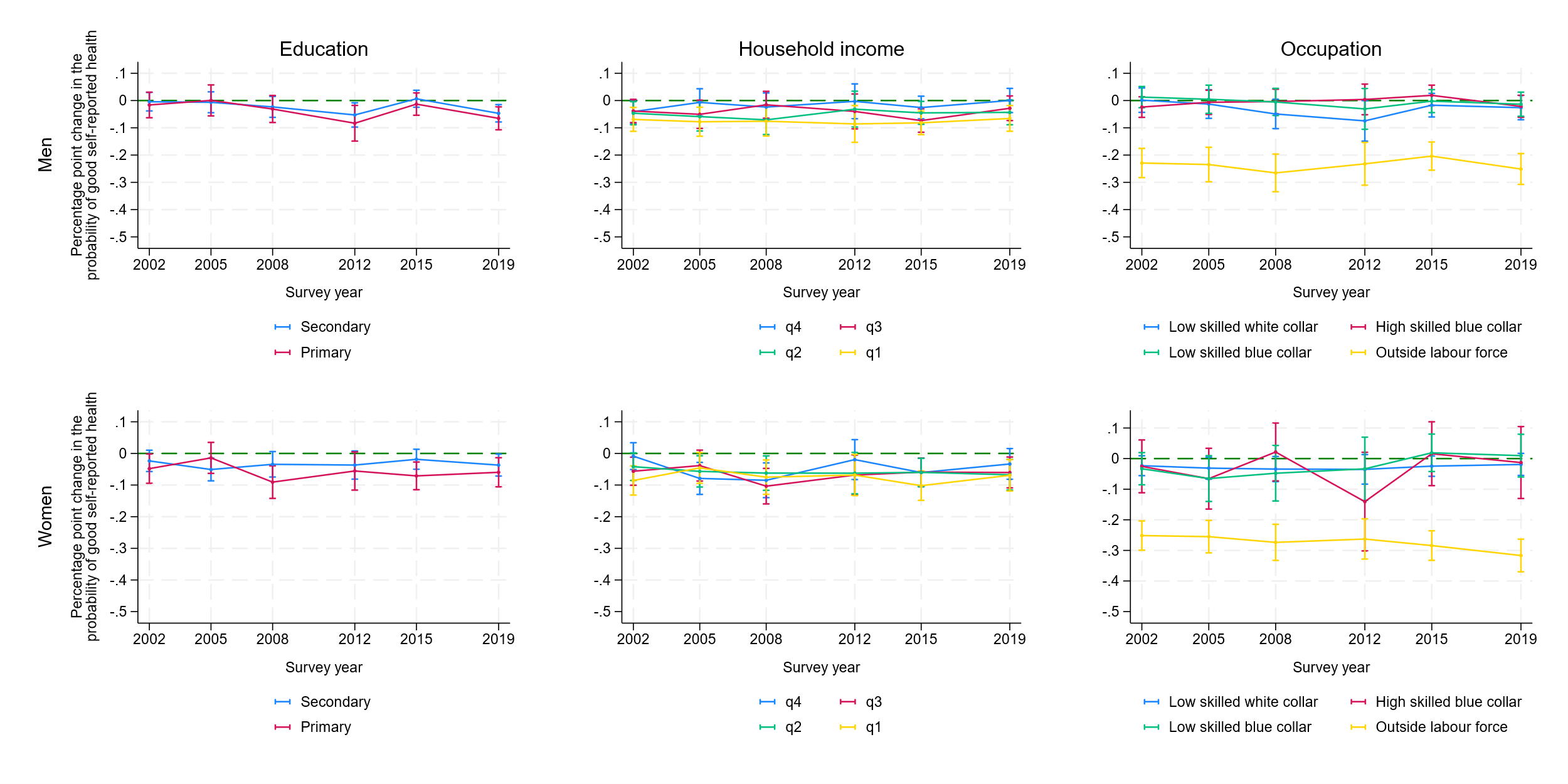


**Figure A4c. Relative (percent) difference (eydx) over time in reports of good health among men and women, by education, income and occupation, Norway 2002-2019, from models including interaction terms between the relevant exposure variable and year**


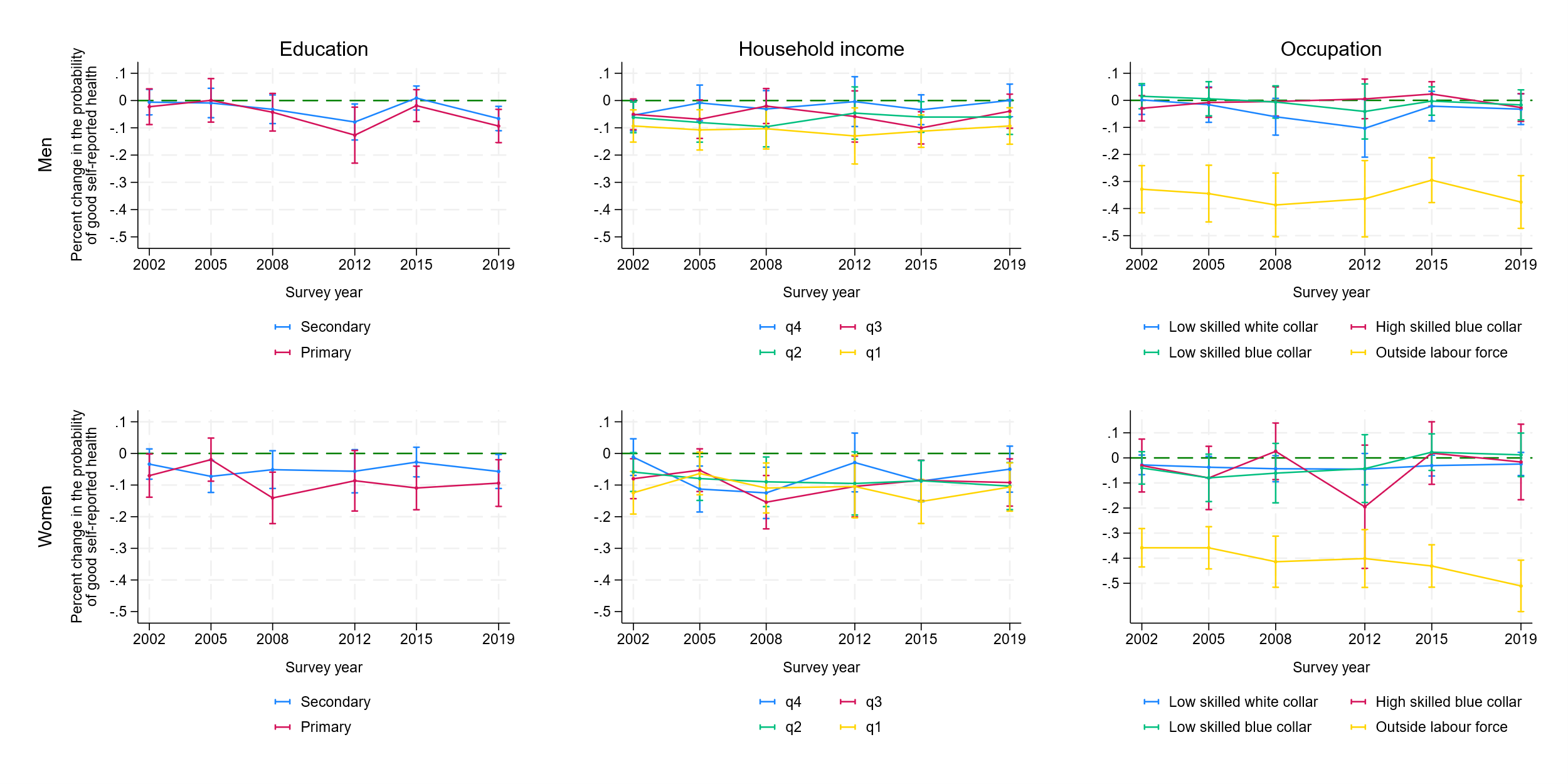


| **Table A4a. Odds ratio (OR) estimates from 3 different models including an interaction term between year and the exposure variables** | | | | | | | | | | | | | | | | | | | |
| --- | --- | --- | --- | --- | --- | --- | --- | --- | --- | --- | --- | --- | --- | --- | --- | --- | --- | --- | --- |
|  |  | **Men** | | | | | | | | | **Women** | | | | | | | | |
|  |  | **Model 4e (year x education)** | | | **Model 4i (year x income)** | | | **Model 4o (year x occupation)** | | | **Model 4e (year x education)** | | | **Model 4i (year x income)** | | | **Model 4o (year x occupation)** | | |
|  |  | **OR** | **SE** | **p** | **OR** | **SE** | **p** | **OR** | **SE** | **p** | **OR** | **SE** | **p** | **OR** | **SE** | **p** | **OR** | **SE** | **p** |
| **Year** | 2002 | 1.000 |  |  | 1.000 |  |  | 1.000 |  |  | 1.000 |  |  | 1.000 |  |  | 1.000 |  |  |
|  | 2005 | 0.892 | 0.166 | 0.540 | 0.874 | 0.217 | 0.587 | 0.877 | 0.136 | 0.398 | 1.161 | 0.219 | 0.429 | 1.110 | 0.281 | 0.680 | 1.137 | 0.212 | 0.491 |
|  | 2008 | 1.125 | 0.218 | 0.543 | 0.971 | 0.248 | 0.909 | 1.094 | 0.177 | 0.578 | 0.849 | 0.150 | 0.355 | 0.938 | 0.239 | 0.804 | 0.760 | 0.128 | 0.104 |
|  | 2012 | 0.789 | 0.145 | 0.198 | 0.527 | 0.128 | 0.008 | 0.612 | 0.090 | 0.001 | 0.723 | 0.126 | 0.064 | 0.682 | 0.175 | 0.135 | 0.681 | 0.115 | 0.023 |
|  | 2015 | 0.859 | 0.143 | 0.360 | 0.939 | 0.215 | 0.784 | 0.824 | 0.111 | 0.151 | 0.819 | 0.127 | 0.199 | 0.922 | 0.211 | 0.724 | 0.788 | 0.120 | 0.117 |
|  | 2019 | 1.068 | 0.183 | 0.703 | 0.701 | 0.155 | 0.108 | 0.851 | 0.117 | 0.242 | 0.697 | 0.107 | 0.018 | 0.662 | 0.146 | 0.061 | 0.653 | 0.097 | 0.004 |
|  |  |  |  |  |  |  |  |  |  |  |  |  |  |  |  |  |  |  |  |
| **Age** | Age (linear) | 0.978 | 0.002 | 0.000 | 0.978 | 0.002 | 0.000 | 0.978 | 0.002 | 0.000 | 0.984 | 0.002 | 0.000 | 0.984 | 0.002 | 0.000 | 0.984 | 0.002 | 0.000 |
|  |  |  |  |  |  |  |  |  |  |  |  |  |  |  |  |  |  |  |  |
| **Civil status** | Married | 1.000 |  |  | 1.000 |  |  | 1.000 |  |  | 1.000 |  |  | 1.000 |  |  | 1.000 |  |  |
|  | Cohabiting | 0.932 | 0.069 | 0.341 | 0.927 | 0.068 | 0.302 | 0.932 | 0.069 | 0.342 | 1.066 | 0.080 | 0.395 | 1.065 | 0.080 | 0.408 | 1.069 | 0.081 | 0.378 |
|  | No partner | 0.821 | 0.053 | 0.002 | 0.818 | 0.053 | 0.002 | 0.823 | 0.053 | 0.003 | 0.917 | 0.061 | 0.193 | 0.915 | 0.061 | 0.185 | 0.921 | 0.062 | 0.217 |
|  |  |  |  |  |  |  |  |  |  |  |  |  |  |  |  |  |  |  |  |
| **Rural/urban area** | >100 000 | 1.000 |  |  | 1.000 |  |  | 1.000 |  |  | 1.000 |  |  | 1.000 |  |  | 1.000 |  |  |
|  | 20 000-100 000 | 1.041 | 0.083 | 0.611 | 1.036 | 0.083 | 0.659 | 1.041 | 0.083 | 0.616 | 0.995 | 0.076 | 0.948 | 0.987 | 0.075 | 0.863 | 0.990 | 0.075 | 0.891 |
|  | 2 000-19 999 | 0.940 | 0.070 | 0.408 | 0.934 | 0.070 | 0.363 | 0.938 | 0.070 | 0.394 | 0.988 | 0.070 | 0.860 | 0.985 | 0.070 | 0.829 | 0.989 | 0.070 | 0.879 |
|  | <2 000 | 0.850 | 0.066 | 0.036 | 0.849 | 0.066 | 0.034 | 0.855 | 0.066 | 0.044 | 0.948 | 0.072 | 0.486 | 0.947 | 0.072 | 0.478 | 0.946 | 0.072 | 0.470 |
|  |  |  |  |  |  |  |  |  |  |  |  |  |  |  |  |  |  |  |  |
| **Adjusted household income quintiles** | q5 (highest) | 1.000 |  |  | 1.000 |  |  | 1.000 |  |  | 1.000 |  |  | 1.000 |  |  | 1.000 |  |  |
|  | q4 | 0.847 | 0.078 | 0.073 | 0.675 | 0.145 | 0.068 | 0.846 | 0.078 | 0.071 | 0.666 | 0.061 | 0.000 | 0.918 | 0.202 | 0.696 | 0.668 | 0.061 | 0.000 |
|  | q3 | 0.689 | 0.062 | 0.000 | 0.689 | 0.148 | 0.082 | 0.690 | 0.062 | 0.000 | 0.590 | 0.053 | 0.000 | 0.601 | 0.126 | 0.015 | 0.589 | 0.053 | 0.000 |
|  | q2 | 0.656 | 0.060 | 0.000 | 0.642 | 0.135 | 0.035 | 0.658 | 0.060 | 0.000 | 0.612 | 0.057 | 0.000 | 0.676 | 0.143 | 0.065 | 0.612 | 0.057 | 0.000 |
|  | q1 (lowest) | 0.541 | 0.051 | 0.000 | 0.536 | 0.111 | 0.003 | 0.543 | 0.051 | 0.000 | 0.533 | 0.053 | 0.000 | 0.482 | 0.100 | 0.000 | 0.534 | 0.053 | 0.000 |
|  |  |  |  |  |  |  |  |  |  |  |  |  |  |  |  |  |  |  |  |
| **Education** | Long | 1.000 |  |  | 1.000 |  |  | 1.000 |  |  | 1.000 |  |  | 1.000 |  |  | 1.000 |  |  |
|  | Medium | 0.963 | 0.148 | 0.806 | 0.845 | 0.058 | 0.014 | 0.846 | 0.058 | 0.014 | 0.814 | 0.125 | 0.181 | 0.768 | 0.052 | 0.000 | 0.768 | 0.052 | 0.000 |
|  | Short | 0.870 | 0.178 | 0.496 | 0.753 | 0.067 | 0.001 | 0.756 | 0.067 | 0.002 | 0.671 | 0.130 | 0.039 | 0.636 | 0.054 | 0.000 | 0.633 | 0.054 | 0.000 |
|  |  |  |  |  |  |  |  |  |  |  |  |  |  |  |  |  |  |  |  |
| **Occupation/Employment** | High-skilled white collar | 1.000 |  |  | 1.000 |  |  | 1.000 |  |  | 1.000 |  |  | 1.000 |  |  | 1.000 |  |  |
|  | Low-skilled white collar | 0.804 | 0.073 | 0.016 | 0.804 | 0.073 | 0.016 | 1.015 | 0.235 | 0.950 | 0.792 | 0.060 | 0.002 | 0.794 | 0.060 | 0.002 | 0.790 | 0.133 | 0.161 |
|  | High-skilled blue collar | 0.942 | 0.079 | 0.480 | 0.943 | 0.079 | 0.488 | 0.800 | 0.140 | 0.202 | 0.771 | 0.145 | 0.166 | 0.782 | 0.147 | 0.191 | 0.775 | 0.320 | 0.537 |
|  | Low-skilled blue collar | 0.969 | 0.093 | 0.742 | 0.972 | 0.093 | 0.767 | 1.143 | 0.246 | 0.535 | 0.807 | 0.108 | 0.109 | 0.804 | 0.107 | 0.102 | 0.722 | 0.179 | 0.190 |
|  | Not employed | 0.235 | 0.018 | 0.000 | 0.237 | 0.018 | 0.000 | 0.226 | 0.036 | 0.000 | 0.188 | 0.014 | 0.000 | 0.188 | 0.014 | 0.000 | 0.190 | 0.030 | 0.000 |
|  |  |  |  |  |  |  |  |  |  |  |  |  |  |  |  |  |  |  |  |
| **Smoking** | Do not smoke | 1.000 |  |  | 1.000 |  |  | 1.000 |  |  | 1.000 |  |  | 1.000 |  |  | 1.000 |  |  |
|  | Occasionally | 1.047 | 0.102 | 0.640 | 1.044 | 0.102 | 0.659 | 1.033 | 0.101 | 0.737 | 1.007 | 0.099 | 0.947 | 1.009 | 0.100 | 0.930 | 1.005 | 0.099 | 0.959 |
|  | Daily | 0.678 | 0.044 | 0.000 | 0.676 | 0.043 | 0.000 | 0.678 | 0.044 | 0.000 | 0.647 | 0.041 | 0.000 | 0.643 | 0.041 | 0.000 | 0.645 | 0.041 | 0.000 |
|  |  |  |  |  |  |  |  |  |  |  |  |  |  |  |  |  |  |  |  |
| **Exercise** | ≥ 1 time/week | 1.000 |  |  | 1.000 |  |  | 1.000 |  |  | 1.000 |  |  | 1.000 |  |  | 1.000 |  |  |
|  | < 1 time/week | 0.710 | 0.051 | 0.000 | 0.710 | 0.051 | 0.000 | 0.706 | 0.051 | 0.000 | 0.812 | 0.064 | 0.009 | 0.814 | 0.064 | 0.009 | 0.813 | 0.064 | 0.009 |
|  | Never | 0.589 | 0.040 | 0.000 | 0.593 | 0.040 | 0.000 | 0.589 | 0.040 | 0.000 | 0.617 | 0.046 | 0.000 | 0.617 | 0.046 | 0.000 | 0.615 | 0.046 | 0.000 |
|  |  |  |  |  |  |  |  |  |  |  |  |  |  |  |  |  |  |  |  |
| **Body Mass Index** | Normal/underweight | 1.000 |  |  | 1.000 |  |  | 1.000 |  |  | 1.000 |  |  | 1.000 |  |  | 1.000 |  |  |
|  | Overweight | 0.772 | 0.046 | 0.000 | 0.768 | 0.046 | 0.000 | 0.774 | 0.046 | 0.000 | 0.708 | 0.041 | 0.000 | 0.709 | 0.041 | 0.000 | 0.709 | 0.041 | 0.000 |
|  | Obesity | 0.342 | 0.026 | 0.000 | 0.341 | 0.026 | 0.000 | 0.340 | 0.025 | 0.000 | 0.360 | 0.027 | 0.000 | 0.362 | 0.027 | 0.000 | 0.363 | 0.027 | 0.000 |
|  |  |  |  |  |  |  |  |  |  |  |  |  |  |  |  |  |  |  |  |
| **People to ask in case of personal trouble?** | 3+ | 1.000 |  |  | 1.000 |  |  | 1.000 |  |  | 1.000 |  |  | 1.000 |  |  | 1.000 |  |  |
|  | 1 or 2 | 0.862 | 0.052 | 0.015 | 0.861 | 0.052 | 0.014 | 0.861 | 0.052 | 0.014 | 0.787 | 0.052 | 0.000 | 0.790 | 0.052 | 0.000 | 0.793 | 0.053 | 0.000 |
|  | No one | 0.831 | 0.168 | 0.359 | 0.818 | 0.165 | 0.319 | 0.831 | 0.168 | 0.359 | 0.680 | 0.169 | 0.121 | 0.668 | 0.167 | 0.106 | 0.672 | 0.167 | 0.110 |
|  |  |  |  |  |  |  |  |  |  |  |  |  |  |  |  |  |  |  |  |
| **Someone to confide in?** | Yes | 1.000 |  |  | 1.000 |  |  | 1.000 |  |  | 1.000 |  |  | 1.000 |  |  | 1.000 |  |  |
|  | No | 0.699 | 0.089 | 0.005 | 0.699 | 0.089 | 0.005 | 0.699 | 0.089 | 0.005 | 0.722 | 0.125 | 0.059 | 0.713 | 0.123 | 0.050 | 0.712 | 0.123 | 0.049 |
|  |  |  |  |  |  |  |  |  |  |  |  |  |  |  |  |  |  |  |  |
| **Year x Education (ref=2002)** | 2005*Long | 1.000 |  |  |  |  |  |  |  |  | 1.000 |  |  |  |  |  |  |  |  |
|  | 2005*Medium | 0.985 | 0.216 | 0.946 |  |  |  |  |  |  | 0.783 | 0.175 | 0.273 |  |  |  |  |  |  |
|  | 2005*Short | 1.155 | 0.360 | 0.645 |  |  |  |  |  |  | 1.301 | 0.389 | 0.379 |  |  |  |  |  |  |
|  | 2008*Long | 1.000 |  |  |  |  |  |  |  |  | 1.000 |  |  |  |  |  |  |  |  |
|  | 2008*Medium | 0.842 | 0.195 | 0.457 |  |  |  |  |  |  | 0.935 | 0.204 | 0.758 |  |  |  |  |  |  |
|  | 2008*Short | 0.874 | 0.258 | 0.646 |  |  |  |  |  |  | 0.773 | 0.201 | 0.322 |  |  |  |  |  |  |
|  | 2012*Long | 1.000 |  |  |  |  |  |  |  |  | 1.000 |  |  |  |  |  |  |  |  |
|  | 2012*Medium | 0.711 | 0.158 | 0.124 |  |  |  |  |  |  | 0.937 | 0.208 | 0.770 |  |  |  |  |  |  |
|  | 2012*Short | 0.649 | 0.191 | 0.142 |  |  |  |  |  |  | 1.002 | 0.282 | 0.994 |  |  |  |  |  |  |
|  | 2015*Long | 1.000 |  |  |  |  |  |  |  |  | 1.000 |  |  |  |  |  |  |  |  |
|  | 2015*Medium | 1.097 | 0.217 | 0.642 |  |  |  |  |  |  | 1.059 | 0.206 | 0.770 |  |  |  |  |  |  |
|  | 2015*Short | 1.036 | 0.266 | 0.891 |  |  |  |  |  |  | 0.884 | 0.213 | 0.610 |  |  |  |  |  |  |
|  | 2019*Long | 1.000 |  |  |  |  |  |  |  |  | 1.000 |  |  |  |  |  |  |  |  |
|  | 2019*Medium | 0.703 | 0.143 | 0.083 |  |  |  |  |  |  | 0.940 | 0.182 | 0.751 |  |  |  |  |  |  |
|  | 2019*Short | 0.680 | 0.176 | 0.136 |  |  |  |  |  |  | 0.982 | 0.238 | 0.939 |  |  |  |  |  |  |
|  |  |  |  |  |  |  |  |  |  |  |  |  |  |  |  |  |  |  |  |
| **Year x Adjusted household income quintiles (ref=2002)** | 2005*q5 |  |  |  | 1.000 |  |  |  |  |  |  |  |  | 1.000 |  |  |  |  |  |
|  | 2005*q4 |  |  |  | 1.388 | 0.459 | 0.322 |  |  |  |  |  |  | 0.534 | 0.174 | 0.054 |  |  |  |
|  | 2005*q3 |  |  |  | 0.931 | 0.297 | 0.824 |  |  |  |  |  |  | 1.136 | 0.370 | 0.695 |  |  |  |
|  | 2005*q2 |  |  |  | 0.937 | 0.295 | 0.836 |  |  |  |  |  |  | 0.863 | 0.277 | 0.646 |  |  |  |
|  | 2005*q1 |  |  |  | 0.978 | 0.300 | 0.942 |  |  |  |  |  |  | 1.330 | 0.421 | 0.368 |  |  |  |
|  | 2008*q5 |  |  |  | 1.000 |  |  |  |  |  |  |  |  | 1.000 |  |  |  |  |  |
|  | 2008*q4 |  |  |  | 1.170 | 0.397 | 0.643 |  |  |  |  |  |  | 0.537 | 0.176 | 0.057 |  |  |  |
|  | 2008*q3 |  |  |  | 1.239 | 0.415 | 0.522 |  |  |  |  |  |  | 0.722 | 0.230 | 0.306 |  |  |  |
|  | 2008*q2 |  |  |  | 0.832 | 0.270 | 0.572 |  |  |  |  |  |  | 0.862 | 0.279 | 0.646 |  |  |  |
|  | 2008*q1 |  |  |  | 0.959 | 0.306 | 0.897 |  |  |  |  |  |  | 1.099 | 0.344 | 0.763 |  |  |  |
|  | 2012*q5 |  |  |  | 1.000 |  |  |  |  |  |  |  |  | 1.000 |  |  |  |  |  |
|  | 2012*q4 |  |  |  | 1.452 | 0.473 | 0.253 |  |  |  |  |  |  | 0.930 | 0.319 | 0.832 |  |  |  |
|  | 2012*q3 |  |  |  | 1.096 | 0.345 | 0.771 |  |  |  |  |  |  | 0.997 | 0.321 | 0.992 |  |  |  |
|  | 2012*q2 |  |  |  | 1.243 | 0.394 | 0.492 |  |  |  |  |  |  | 0.922 | 0.305 | 0.806 |  |  |  |
|  | 2012*q1 |  |  |  | 1.059 | 0.325 | 0.851 |  |  |  |  |  |  | 1.244 | 0.400 | 0.497 |  |  |  |
|  | 2015*q5 |  |  |  | 1.000 |  |  |  |  |  |  |  |  | 1.000 |  |  |  |  |  |
|  | 2015*q4 |  |  |  | 1.156 | 0.345 | 0.627 |  |  |  |  |  |  | 0.646 | 0.193 | 0.144 |  |  |  |
|  | 2015*q3 |  |  |  | 0.769 | 0.223 | 0.363 |  |  |  |  |  |  | 0.997 | 0.287 | 0.991 |  |  |  |
|  | 2015*q2 |  |  |  | 1.026 | 0.297 | 0.930 |  |  |  |  |  |  | 0.881 | 0.255 | 0.662 |  |  |  |
|  | 2015*q1 |  |  |  | 0.929 | 0.260 | 0.792 |  |  |  |  |  |  | 0.916 | 0.256 | 0.753 |  |  |  |
|  | 2019*q5 |  |  |  | 1.000 |  |  |  |  |  |  |  |  | 1.000 |  |  |  |  |  |
|  | 2019*q4 |  |  |  | 1.485 | 0.435 | 0.177 |  |  |  |  |  |  | 0.840 | 0.247 | 0.553 |  |  |  |
|  | 2019*q3 |  |  |  | 1.149 | 0.329 | 0.628 |  |  |  |  |  |  | 1.059 | 0.298 | 0.839 |  |  |  |
|  | 2019*q2 |  |  |  | 1.101 | 0.309 | 0.732 |  |  |  |  |  |  | 0.897 | 0.251 | 0.699 |  |  |  |
|  | 2019*q1 |  |  |  | 1.126 | 0.310 | 0.666 |  |  |  |  |  |  | 1.244 | 0.341 | 0.425 |  |  |  |
|  |  |  |  |  |  |  |  |  |  |  |  |  |  |  |  |  |  |  |  |
| **Year x Occupation/ Employment (ref=2002)** | 2005*HSWC |  |  |  |  |  |  | 1.000 |  |  |  |  |  |  |  |  | 1.000 |  |  |
|  | 2005*LSWC |  |  |  |  |  |  | 0.879 | 0.287 | 0.694 |  |  |  |  |  |  | 0.906 | 0.231 | 0.698 |
|  | 2005*HSBC |  |  |  |  |  |  | 1.179 | 0.313 | 0.535 |  |  |  |  |  |  | 0.685 | 0.401 | 0.518 |
|  | 2005*LSBC |  |  |  |  |  |  | 0.915 | 0.298 | 0.785 |  |  |  |  |  |  | 0.737 | 0.296 | 0.447 |
|  | 2005*Not employed |  |  |  |  |  |  | 1.040 | 0.247 | 0.869 |  |  |  |  |  |  | 0.912 | 0.219 | 0.700 |
|  | 2008*HSWC |  |  |  |  |  |  | 1.000 |  |  |  |  |  |  |  |  | 1.000 |  |  |
|  | 2008*LSWC |  |  |  |  |  |  | 0.628 | 0.206 | 0.156 |  |  |  |  |  |  | 0.951 | 0.225 | 0.832 |
|  | 2008*HSBC |  |  |  |  |  |  | 1.204 | 0.356 | 0.529 |  |  |  |  |  |  | 1.596 | 1.040 | 0.473 |
|  | 2008*LSBC |  |  |  |  |  |  | 0.829 | 0.280 | 0.580 |  |  |  |  |  |  | 0.941 | 0.394 | 0.884 |
|  | 2008*Not employed |  |  |  |  |  |  | 0.801 | 0.201 | 0.377 |  |  |  |  |  |  | 1.044 | 0.238 | 0.850 |
|  | 2012*HSWC |  |  |  |  |  |  | 1.000 |  |  |  |  |  |  |  |  | 1.000 |  |  |
|  | 2012*LSWC |  |  |  |  |  |  | 0.607 | 0.197 | 0.123 |  |  |  |  |  |  | 0.962 | 0.239 | 0.875 |
|  | 2012*HSBC |  |  |  |  |  |  | 1.288 | 0.352 | 0.355 |  |  |  |  |  |  | 0.514 | 0.313 | 0.274 |
|  | 2012*LSBC |  |  |  |  |  |  | 0.706 | 0.234 | 0.293 |  |  |  |  |  |  | 1.065 | 0.488 | 0.891 |
|  | 2012*Not employed |  |  |  |  |  |  | 1.230 | 0.309 | 0.410 |  |  |  |  |  |  | 1.157 | 0.275 | 0.538 |
|  | 2015*HSWC |  |  |  |  |  |  | 1.000 |  |  |  |  |  |  |  |  | 1.000 |  |  |
|  | 2015*LSWC |  |  |  |  |  |  | 0.853 | 0.249 | 0.587 |  |  |  |  |  |  | 1.021 | 0.221 | 0.924 |
|  | 2015*HSBC |  |  |  |  |  |  | 1.495 | 0.373 | 0.106 |  |  |  |  |  |  | 1.512 | 1.041 | 0.549 |
|  | 2015*LSBC |  |  |  |  |  |  | 0.859 | 0.243 | 0.591 |  |  |  |  |  |  | 1.673 | 0.680 | 0.205 |
|  | 2015*Not employed |  |  |  |  |  |  | 1.243 | 0.263 | 0.305 |  |  |  |  |  |  | 0.978 | 0.201 | 0.912 |
|  | 2019*HSWC |  |  |  |  |  |  | 1.000 |  |  |  |  |  |  |  |  | 1.000 |  |  |
|  | 2019*LSWC |  |  |  |  |  |  | 0.792 | 0.233 | 0.428 |  |  |  |  |  |  | 1.088 | 0.234 | 0.694 |
|  | 2019*HSBC |  |  |  |  |  |  | 1.043 | 0.251 | 0.862 |  |  |  |  |  |  | 1.164 | 0.725 | 0.807 |
|  | 2019*LSBC |  |  |  |  |  |  | 0.778 | 0.222 | 0.379 |  |  |  |  |  |  | 1.504 | 0.589 | 0.297 |
|  | 2019*Not employed |  |  |  |  |  |  | 0.976 | 0.212 | 0.913 |  |  |  |  |  |  | 0.927 | 0.192 | 0.715 |
| **Constant** |  | 63.081 | 12.223 | 0.000 | 73.490 | 15.883 | 0.000 | 68.823 | 12.318 | 0 | 54.857 | 10.112 | 0.000 | 52.800 | 10.895 | 0.000 | 56.384 | 10.079 | 0.000 |
| **BIC** |  | 10122 |  |  | 10216 |  |  | 10214 |  |  | 10284 |  |  | 10367 |  |  | 10376 |  |  |
| **AIC** |  | 9818 |  |  | 9838 |  |  | 9835 |  |  | 9981 |  |  | 9989 |  |  | 9998 |  |  |
| **N** |  | 12293 |  |  | 12293 |  |  | 12293 |  |  | 12130 |  |  | 12130 |  |  | 12130 |  |  |

1. Formally, in Stata the dydx-command calculates the probability point change in y with one unit change in x, while the eydx-command calculates the percent change in ln(y) with one unit change in x. [↑](#footnote-ref-1)
