# Supplementary material for: Changes over time in social inequality in adult self-rated health: the case of Norway 2002–2019
Source: BMC Public Health. 2025 Nov 11;25:3894. doi: 10.1186/s12889-025-25248-w (PMC12606821; doi:10.1186/s12889-025-25248-w)
Supplement: Supplementary file 5 — Additional file 5. Relative Index of Inequality (RII) and Slope Index of Inequality (SII). Figure A5a. Relative index of inequality (RII) over time in reports of good health from a modified Model 4, based on ridits for education, income and occupation, Norway 2002-2019. Figure A5b. Slope index of inequality (SII) over time in reports of good health from Model 4 (men) and Model 3 (women), based on ridits for education, income and occupation, Norway 2002-2019. [file 12889_2025_25248_MOESM5_ESM.docx]

# Additional file 5: Relative Index of Inequality (RII) and Slope Index of Inequality (SII)

In these analyses, we employed ridits. In short, a *ridit* analysis transforms a categorial variable (for example *Education*) to a numeric variable from which one can calculate RII (the relative increase from the lowest to the highest value) and SII (the absolute difference between the lowest to the highest value). In the Stata documentation for the *wridit*-command, ridits are defined as: “Given a variable X, the unfolded ridit for a value x is equal to the probability that X<x plus half the probability that X==x.” (e.g., the marginal distribution of the dependent variable). For a brief explanation of ridits, cf. Strand et al. (2014). For a detailed description, cf. Harper and Lynch (2006); Newson (2024).

RII and SII were calculated by regressing the transformed *Education*, *Income* and *Occupation* variables on all variables using a Generalized Linear Model with *family(poisson) link(log)* in the case of RII^[[1]](#footnote-1)^, and *family (poisson) link(identity)* in the case of SII.

In short, we find that the trends are remarkably stable over time, in both absolute and relative terms.

**Figure A5a. Relative index of inequality (RII) over time in reports of good health from a modified Model 4, based on ridits for education, income and occupation, Norway 2002-2019**


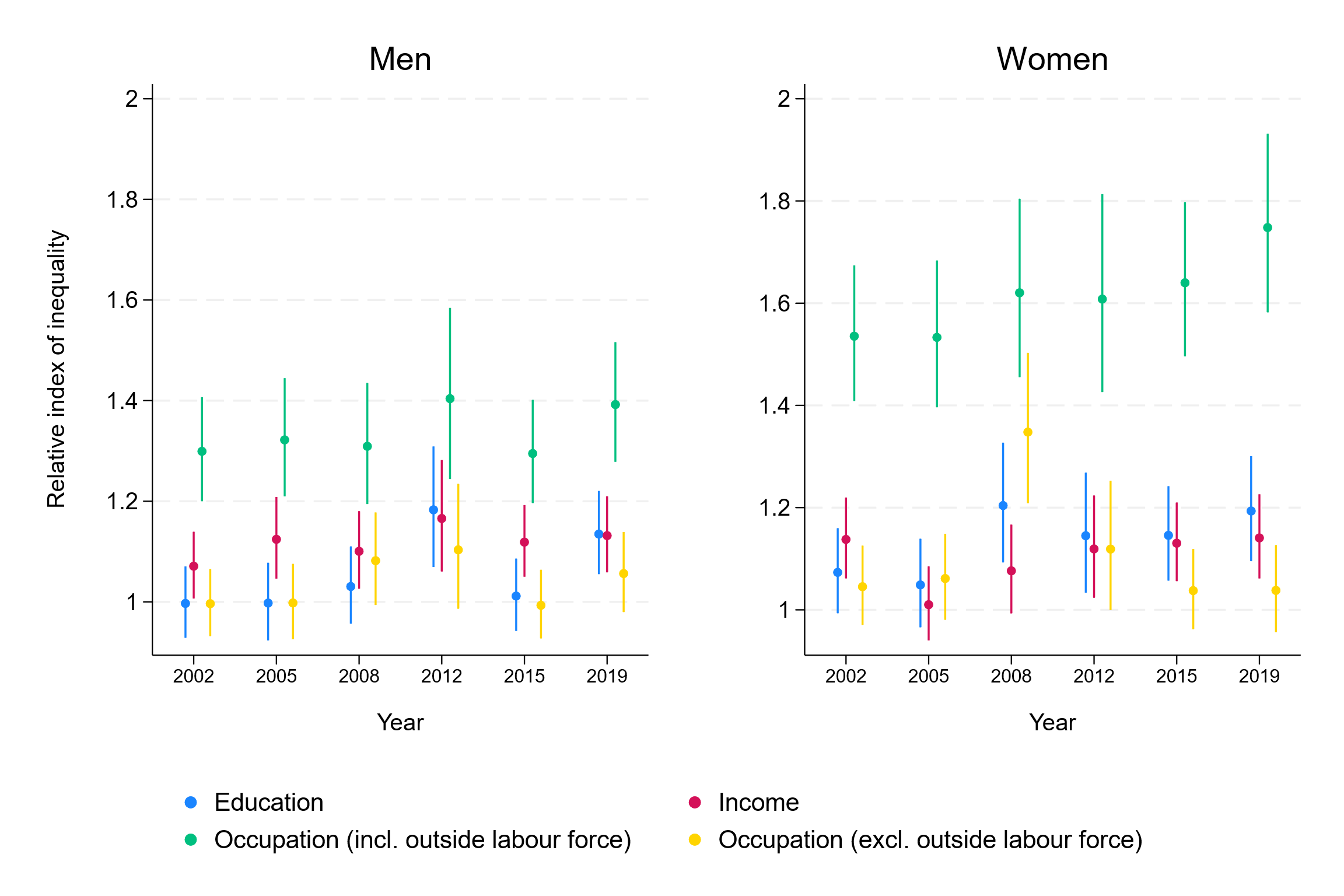


**Figure A5b. Slope index of inequality (SII) over time in reports of good health from Model 4 (men) and Model 3 (women), based on ridits for education, income and occupation, Norway 2002-2019^[[2]](#footnote-2)^**


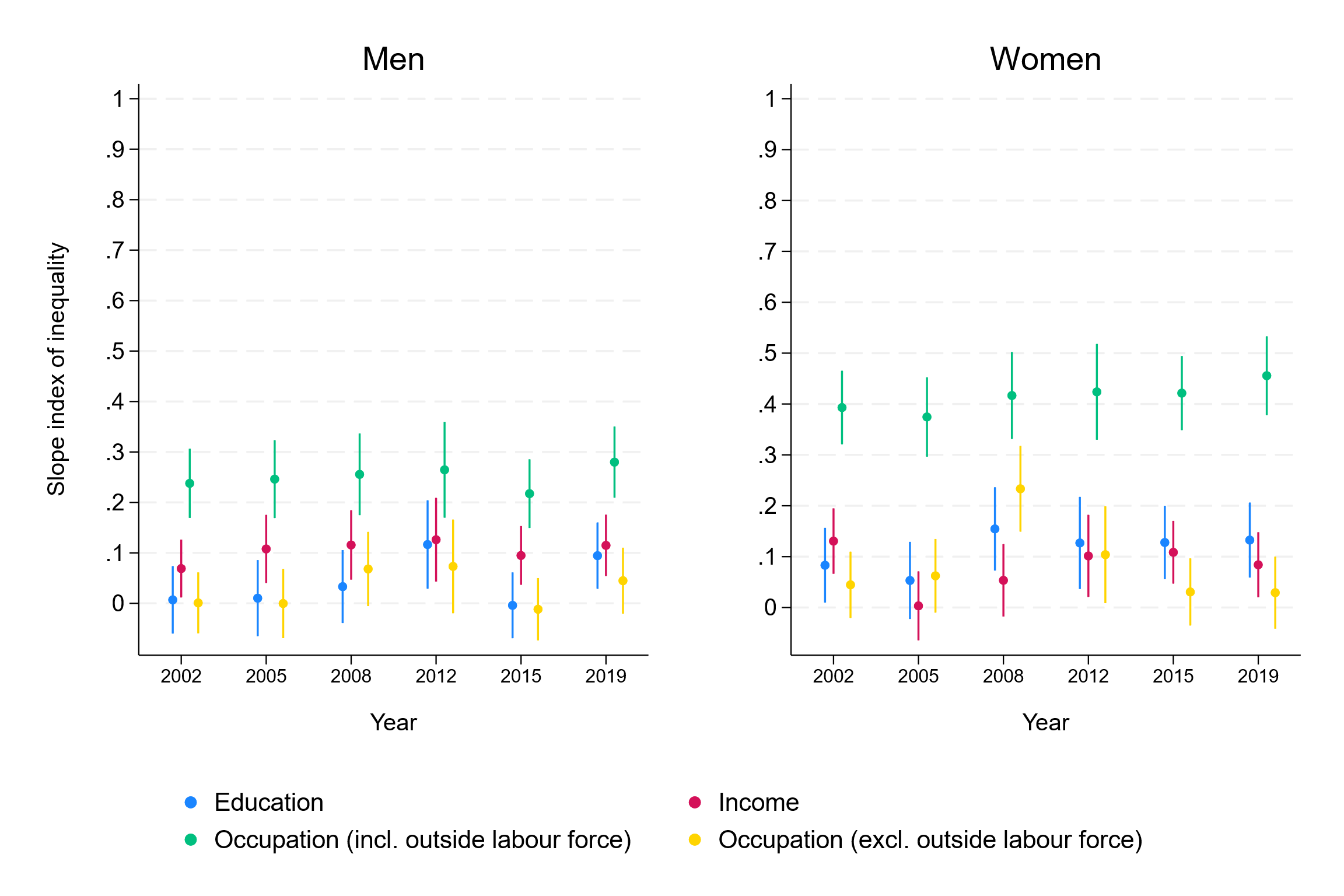


# References

Harper S. & Lynch, J. *Measuring health inequalities. Methods in Social Epidemiology*. Edited by: Oakes, J.M. & Kaufman, J.S. 2006, San Francisco, USA: Jossey-Bass.

Newson R. Generate weighted ridits 2024 [Available from: http://fmwww.bc.edu/RePEc/bocode/w/wridit.sthlp].

Strand, B.H., Steingrímsdóttir, Ó.A., Grøholt, EK. *et al.* Trends in educational inequalities in cause specific mortality in Norway from 1960 to 2010: a turning point for educational inequalities in cause specific mortality of Norwegian men after the millennium? *BMC Public Health* **14**, 1208 (2014). <https://doi.org/10.1186/1471-2458-14-1208>

1. RII is the exponentiated coefficient from this model. [↑](#footnote-ref-1)
2. Model 4 did not converge for women, and the estimates for women are therefore based on Model 3. [↑](#footnote-ref-2)
